# An improved immunoassay detects Aβ oligomers in human biofluids: their CSF levels rise with tau and phosphotau levels

**DOI:** 10.1186/s13195-025-01802-x

**Published:** 2025-07-12

**Authors:** Ting Yang, Yi Ran Xu, Shanxue Jin, Nagendran Ramalingam, Jean-Pierre Bellier, Alexandra M. Lish, Beth L. Ostaszewski, Tracy Young-Pearse, Lei Liu, Hyun-Sik Yang, Jasmeer P. Chhatwal, Trebor L. Lawton, Dennis J. Selkoe

**Affiliations:** 1 Department of Neurology, Ann Romney Center for Neurologic Diseases, Brigham and Women’s Hospital, Harvard Medical School, 60 Fenwood Road, Boston, MA 02115 USA; 2 Center for Alzheimer Research and Treatment, Department of Neurology, Brigham and Women’s Hospital, Harvard Medical School, Boston, USA; 3 Abyssinia Biologics, Inc, Durham, NH USA

**Keywords:** Alzheimer’s disease, Biomarkers, Amyloid β-protein, Oligomeric Aβ, Tau, Monitoring

## Abstract

**Background:**

Diffusible Aβ oligomers (oAβ) confer cytotoxicity in Alzheimer’s disease. The dynamic complexity of this hydrophobic analyte means few immunoassays exist to quantify oAβ in CSF and plasma.

**Methods:**

We characterized antibody 71A1 to a cyclized dimer of Aβ9-18 for oAβ preference over monomers by surface plasmon resonance. We improved an earlier bead-based immunoassay by using 71A1 streptavidin plates for capture and N-terminal antibody 3D6 for detection. Numerous controls systematically validated accuracy.

**Results:**

71A1 showed highly selective binding kinetics to Aβ oligomers over monomers. It enriched bioactive oligomers from AD brain that altered neuronal excitatory currents and calcium transients. 71A1/3D6 immunoassay exhibited specificity and reproducibility in human biofluids. CSF oAβ levels correlated positively with CSF tau and phosphorylated-tau-181. APP and PS1 FAD mutations increased oAβ levels in human neuronal media.

**Conclusions:**

CSF oAβ levels rise in concert with rising tau levels. A new plate-based ELISA offers improved consistency, less sample volume, and lower cost, thus better suited to quantify this challenging analyte.

**Supplementary Information:**

The online version contains supplementary material available at 10.1186/s13195-025-01802-x.

## Introduction

The ability of soluble oligomers of amyloid β-peptide (Aβ) to confer neurotoxicity and related glial inflammation in Alzheimer's disease (AD) has become increasingly evident [1–3]. Aqueously infusible Aβ oligomers (oAβ) play key roles in inducing tau-positive neuritic dystrophy, microgliosis, and associated functional impairments in various Alzheimer-relevant cellular and animal models [4–7]. Their synaptotoxicity and associated microglial activation support their pathogenic role, recommending these diffusible species as potential biomarkers for diagnosis and monitoring as well as promising therapeutic targets [8–12]

Despite the widely reported mechanistic evidence of a role for oAβ, detecting and quantifying these hydrophobic, highly variable assemblies in biological samples is challenging. Although antibodies with preferential binding to Aβ oligomers over monomers have been developed, quantifying the small amounts of naturally occurring oAβ in cerebrospinal fluid (CSF) and plasma poses technical hurdles [13–15]. Moreover, oligomers are self-aggregating and have been shown to change readily during the dynamic processes of Ab42 aggregation and disaggregation [16]. Recently, we developed a bead-based assay that employs the conformational Aβ antibody 71A1 as capture and the Asp1-specific 3D6 antibody as detector. It showed good performance [17] in quantifying oligomers in CSF, but we subsequently found that the bead-conjugated antibody showed inconsistency in some matrixes such as plasma.

71A1 was raised to a cyclized version of two Aβ 9–18 synthetic peptides that was postulated to mimic a dimeric conformation of the N-terminal region, conferring binding preference for oligomeric over monomeric Aβ [17]. 71A1 labels Aβ plaques in unfixed AD brain sections and has been shown to effectively neutralize the synaptotoxicity of AD brain-derived, aqueously diffusible oAβ [17].

The current study addressed five key goals. First, we established the specificity of 71A1 for Aβ oligomers over monomers using surface plasmon resonance (SPR). Second, we used 71A1 to immunopurified endogenous Aβ oligomers from aqueous extracts of AD cortex and perform whole-cell patch clamp recordings to show they abnormally elevate neuronal excitability and alter calcium transients in primary rodent neurons. Third, we created and validated an improved, plate-based immunoassay using 71A1 for capture and antibody 3D6 for detection. Fourth, we confirmed the assay’s preference for aggregated over monomeric Aβ in human CSF and plasma. Fifth, we established that the levels of natural oAβ in human CSF correlate positively with the levels of tau and phospho-tau.

Our previous bead-based assay format using 71A1 and 3D6 to detect oligomeric Aβ in brain extracts, CSF, and plasma helped establish the principle of a sandwich immunoassay for quantifying diffusible oAβ. [17]. However, subsequent scaling of the bead-based assay to assess patient cohorts presented unanticipated technical challenges: we observed variability among production lots of beads that introduced anomalous behavior, with some bead lots exhibiting high coefficients of variation (CV) and others displaying non-specific binding in aqueous brain extracts and plasma, although not in CSF. This variability of bead lots compromised the stability and consistency of the bead-based assay results, especially in analyzing larger cohorts with heterogeneous patient samples that require uniform reagents obtained in bulk. This necessitated rigorous evaluation of different reagent batches, leading to challenging reagent procurement requirements and excess time for assay preparation before runs.

To overcome the technical limitations of the bead-based assay and make it practical for potential diagnostic and therapeutic monitoring, we have undertaken extensive troubleshooting experiments to transition the original bead-based assay to a far more consistent plate-based platform. The new protocol offers comparable detection sensitivity with improved assay efficiency, requiring less biological sample volume than previously needed for the bead-based assay [17]. The plate-based assay workflow is more condensed for ease of execution and eliminates dependency on varying bead lots. The upgraded assay also offers substantial cost savings, reducing expenses to < 70% of the original bead-based method. Collectively, these improvements make the plate-based 71A1/3D6 assay a significantly more reliable platform to assess oAβ levels in larger patient populations.

## Methods

### Reagents and antibodies

Amyloid-β derived diffusible ligands (ADDLs) were prepared from Aβ1-42 synthetic peptide (Yale University New Haven, CT, USA) as per previous reports [1]. Monoclonal antibody (mAb) 3D6 is highly specific for the extreme N-terminus (Asp-1) of human Aβ [18]. Monoclonal antibody 71A1 was raised against a synthetic conformational peptide immunogen consisting of two peptides of residues 9–18 of human Aβ. These amino acid chains are cyclized, and the resulting dimer-like peptide consist of residues in this region that associate into a three-dimensional configuration mimicking the structure of aggregated polymers of Aβ [19].

### Human study participant details and experimental AD mouse model

Human CSF and plasma samples were obtained from the BWH Division of Cognitive and Behavioral Neurology. Supplementary Table 1 presents the demographic characteristics and further details of the cohorts that were used for assay validation. Information for two of the 108 subjects (including gender) was not available. BWH patients referred for diagnostic lumbar puncture were consented for donation of a plasma sample and extra volume of CSF for research purposes. Access to their medical records was allowed under BWH IRB approval. Blood was collected into EDTA tubes (Becton Dickinson), centrifuged at 1500 g in a tabletop centrifuge for 15 min at 4 °C, and the plasma supernatant was collected, aliquoted and bio-banked at − 80 °C, all within 3 h of collection. CSF was drawn directly into polypropylene tubes (Sarstedt). A portion of CSF was sent to Athena Diagnostics for the ADmark Panel or to Mayo Clinic Laboratories (Rochester, MN) for the ADEVL Test (consisting of Aβ1–42, total-tau, and phosphotau_181_ levels). The remaining volume was frozen immediately on dry ice, then later thawed and aliquoted to be stored at − 80 °C. Clinical diagnostic information was obtained through chart review by a board-certified behavioral neurologist before the ADmark, Mayo, or our research ELISA diagnoses were returned.

Human brain tissue was obtained at BWH or MGH from deceased donors with probable AD undergoing diagnostic autopsy. One hemisphere was fixed for diagnostic purposes and the other hemisphere sliced coronally (~ 3 cm) and frozen at − 80 °C. All human research subjects received prior approval from the Mass General Brigham Institutional Review Board, and informed consent was obtained for all living human participants.

All mouse and rat procedures were approved by the Institutional Animal Care and Use Committee at BWH (IACUC protocol # 2016N000342 and 2016N000305). Mouse hippocampal slice recordings used wild-type (wt) C57Bl/6 mice aged 1–3 mo from Jackson Labs. The APP (NL-G-F) knock-in mice were generously provided by Dr. Takaomi Saido from RIKEN Center for Brain Science, Tokyo, Japan [20].The primary reason this AD mouse model was chosen was to avoid potential artifacts introduced by tg APP overexpression by using a knock-in approach to express APP at wild-type levels under the endogenous promoter. The NL-G-F line also shows cognitive impairment earlier than other typical AD tg mice models [21]. Homozygous mice were monogamously mated to produce offspring, which were housed until sacrificed at the designated age. Mice were anesthetized by isoflurane overdose and subsequent trans-cardiac perfusion with heparinized saline, followed by 4% paraformaldehyde in 0.1 M phosphate buffered saline (PBS). Brains were removed and post-fixed overnight in 4% paraformaldehyde in 0.1 M PBS, followed by equilibration in 15% sucrose in PBS and then 30% sucrose in PBS. Fixed brains were embedded in Tissue-Tek® O.C.T. Compound and frozen at − 80 °C overnight. Frozen blocks were changed to − 20 °C for 2 h to soften tissue for sectioning. Brains were coronally sectioned on a Leica cryostat cm1850 (Wetzlar, Germany) at 25 μm thickness at −19 °C and stored in cryoprotectant at −20 °C until immunohistochemical processing.

### Preparation of aqueous soaking extracts of AD cortex

The soaking method for aqueous extraction of diffusible Aβ aggregates from AD brain has been described [8, 22, 23]. It purposely avoids brain homogenization that would break up fibrillar plaques and vascular amyloid. Grey matter was dissected from fresh or frozen cortex (pooled tissue from frontal, parietal, and temporal lobes) of individuals with neuropathologically confirmed AD and minced using a McIlwain tissue chopper (razor blade) set at 0.05 mm. The resultant brain bits (without homogenization) were soaked for just 30 min in a 5 × ratio of TBS extraction buffer (25 mM Tris supplemented with 150 mM NaCl and protease inhibitors (5 mg/mL leupeptin, 5 mg/mL aprotinin, 2 mg/mL pepstatin, 120 mg/mL 4-benzenesulfonyl fluoride hydrochloride, and phosphatase inhibitor 5 mM NaF, pH 7.2, Roche) in 50-mL Eppendorf Protein Lo-Bind tubes, and then spun at 2,000 g in a Fiberlite F14-14 × 50cy rotor in a Sorvall Lynx 4000 centrifuge for 10 min at 4 °C. TBS was chosen because of its physiologic pH and salt concentration. We chose 0.15 M NaCl TBS over artificial CSF because the latter is not as well buffered. The top 90% of the supernatants were transferred to thin-wall polypropylene tubes (Beckman catalog #331,372) and spun in an SW41Ti rotor at 40,000 rpm for 110 min in an Optima L90K ultracentrifuge at 4 °C (pelleting distance ~ 9 cm). The top ~ 90% of supernatants was retained and frozen at −80 °C in 1 mL aliquots.

### Surface plasmon resonance (SPR)

SPR analysis was performed to compare the interactions of synthetic Aβ40 monomers versus crosslinked synthetic Aβ(S26C)_2_ dimers with immobilized antibodies using the P4PRP and Affipump devices (Affinité Instruments, Montreal, Canada) equipped with amino-coated sensors (Afficoat sensors, Affinité Instruments). For each experiment, sensors were first equilibrated with 1 mL of running buffer (RB: PBS 1X + 0.05% Tween 20). Once baseline stabilization was achieved, the amino groups on the sensor were activated with 1:1 mix of 30 mM 1-ethyl-3-(3-dimethylaminopropyl) carbodiimide and 100 mM N-hydroxy succinimide for 6 min. After brief rinse with RB, antibody coupling was carried out by injecting the antibody solution (0.02 mg/mL in 110 mM sodium acetate, pH5.0) and allowing binding for 10 min. To block unreacted, activated amino groups on the sensor surface, 1 M ethanolamine (pH 8.5) was injected, and the sensors were washed with RB to ensure stable baseline signals. Ligand binding assays were conducted in kinetic mode by injecting 33 nM Aβ40 monomers or (S26C)_2_ dimers at a flow rate of 480 ml/min for 10 min. Regeneration of the sensor surfaces was performed using a 10 mM glycine–HCl solution (pH 2.5) followed by additional RB washes. Data were collected and analyzed using a 1:1 kinetic model fit (with or without diffusion correction) to evaluate the interaction parameters of the ligands to the immobilized antibody. The quality of the fit was assessed using the Chi2 value, indicating the deviation between the measured and fitted curves (lower is better), and the U-value, which reflects the precision of the kinetic fit (lower is better).

### Electrophysiology recordings

Whole-cell patch-clamp current recordings from rat primary hippocampal neurons at DIV 16–19 were obtained at 32–35 °C in the external solution (in mM): NaCl 145, KCl 5, HEPES 10, glucose 10, MgCl2 2, CaCl2 2 (pH 7.3) and the internal solution (in mM): CsMeSO4 110, NaMeSO4 10, EGTA 10, CaCl2 1, HEPES 10, TEA 10, QX-314 5, MgATP 5, Na2GTP 0.5 (pH 7.2). Hippocampal neurons were prepared as described previously [24]. Spontaneous excitatory postsynaptic currents (sEPSCs) and spontaneous inhibitory postsynaptic currents (sIPSCs) were recorded in voltage clamp mode, holding cells at − 60 and 10 mV, respectively. Recordings were performed with a Multiclamp 700B amplifier (Molecular Devices, San Jose, CA). Signals were filtered at 2 kHz and sampled at 10 kHz with Digidata 1440 A (Molecular Devices, San Jose, CA). Access resistance (Ra) was monitored following membrane rupture and dialysis, and recordings were abandoned if Ra was > 15 MΩ. A pCLAMP 10.2 (Molecular Devices, San Jose, CA) was used for data display, acquisition and storage, and offline analysis of sEPSCs and sIPSCs was performed using the Mini Analysis software. Statistical analyses were performed using GraphPad Prism 7. Data were presented as means ± SEMs. Significant differences were determined using one-way ANOVA test with post hoc Tukey's test or unpaired student’s t-test.

#### Calcium imaging

Calcium transients in DIV16-19 rat cortical neurons treated with 71A1 affinity purified Aβ oligomers for 2 or 5 min were recorded using a Leica DMi8 widefield fluorescence microscope equipped with 5% CO_2_. Cortical neuron culture for calcium imaging was prepared as described previously [24]. Time series images were acquired at 250 ms/frame for 2 min at 37 °C. A customized FIJI ImageJ macro was written to analyze acquired time series data as described [24]. Briefly, the analysis process had two steps: (A) Identification of cells with signal change during the time series: raw data went through smoothing (mean filter, radius 2) and stack projection (standard deviation), then the auto-threshold algorithm “Triangle” was applied to create a mask image of positive cells. The “Watershed” method was used to separate touching cells, and cell ROIs were selected by the “Analyze Particle” function in ImageJ. (B) For each cell ROI selected by step A, a profile of mean fluorescence intensity inside the ROI was plotted as a 1D image, the peak positions were identified by the “Find Maxima” function, and false peaks due to noise were removed. The peak positions of each individual cell ROI selection were saved as an Excel file. Finally, the correlation of peak positions among all cell ROIs was measured (the correlation was defined as how compact the peak position clusters were). Codes used for the analysis are available in a GitHub repository (https://github.com/udettmer/dettmerlab).

#### Sequential immunoprecipitation (IP)

1 mL of 4 × diluted human brain TBS soaking extracts was incubated with 20 μg of the indicated antibodies and nutated for 1 h at 4 °C. The immunoprecipitated (IP) solution was then further incubated with Protein G Magnetic Dynabeads (Invitrogen), MyOne C1 Streptavidin Dynabeads (Fisher Scientific) or in Streptavidin Coated High-Capacity Plates (Pierce) overnight at 4 °C, followed by three washes with TBS (Bio-Rad 20 mM TRIS, 0.5 M NaCl, pH 7.5) supplemented with 0.02% TWEEN-20. Post-IP supernatants were subjected to three additional immunoprecipitations using fresh beads/plates and antibodies, and the final supernatants were saved for downstream analysis.

#### Western blotting (WB)

(S26C)_2_ dimeric Ab peptides (Bachem) were left untreated or exposed to 10% 2-Mercaptoethanol (βME) overnight undisturbed at 4 °C. Samples were electrophoresed as described [25] using 26-well 4–12% bis–Tris gels and MES running buffer (Invitrogen) [12]. After electrophoresis, gels were transferred to 0.2 μm nitrocellulose membranes, boiled for 90 s on each side, and reacted with a cocktail consisting of 1 mg/mL of anti-Ab40 (mAb 2G3) and anti-Ab42 (mAb 21F12) and 0.5 mg/mL of Ab N-terminal antibody (6E10) overnight in 2 × diluted Intercept blocking buffer (Li-Cor) in TBS at 4 °C. The membrane was then incubated with 20,000 × diluted Alexa Fluor-647 conjugated anti-mouse secondary antibodies (AbCam) in 2 × diluted blocking buffer in TBS. Immunofluorescence was visualized on a Li-Cor Imaging Reader.

#### Induced pluripotent stem cell (iPSC) lines

iPSC familial Alzheimer’s disease (FAD) lines were obtained from NYSCF and are previously described [26]. CRISPR/Cas9 gene editing was employed to knock in certain FAD-causing mutations: *APP*^*swe*^*, PSEN1*^*M146V*^*,* and *APP *^*Icelandic*^*.* The isogenic iPSC lines utilized in this study include a complex heterozygous line for both *APP*^*swe*^ and *PSEN1*^*M146V*^*,* a heterozygous line for *APP*^*swe*^*,* a homozygous line for *APP *^*Icelandic*^ (A673T), and an isogenic WT control line (Coriell Institute, catalog ID: AG07889). iPSC lines were utilized following IRB review and approval through MGB/BWH IRB (#2015P001676). iPSCs were reprogrammed as described [27]. iPSCs were maintained using StemFlex Medium (Thermo Fisher Scientific). All cell lines were routinely tested for mycoplasma using PCR kit (MP0035-1KT) and STR profiling to prevent potential contamination or alteration to the cell lines.

#### Differentiation of iPSCs to induced neurons (iNs)

iPSC-derived neurons (iNs) were differentiated as reported [28] with minor modifications [29]. iPSCs were plated at a density of 95 k cells/cm^2^ on plates coated with growth factor reduced Matrigel one day prior to virus transduction (Corning #354,230). Then, iPSCs were transduced with two lentiviruses – pTet-O-NGN2-puro (Addgene plasmid #52,047, a gift from Marius Wernig) and FUdeltaGW-rtTA (Addgene plasmid #19,780, a gift from Konrad Hochedlinger). The cells were then replated at 200 k cells/cm^2^ using StemFlex Medium (Thermo Fisher Scientific) and ROCK inhibitor (10 mM) (day 0). The media was changed to KSR media (day 1), 1:1 of KSR and N2B media (day 2) and N2B media (day 3). Doxycycline (2 μg/mL, Sigma) was added from day 1 to the end of the differentiation, and puromycin (5 mg/mL, Gibco) was added from day 2 to the end of the differentiation. On day 3, B27 supplement (1:100) (Life Technologies) was added. On day 4, cells were dissociated using accutase (diluted 1:3 in PBS) and cryopreserved in a 1:1 ratio of 20% DMSO in FBS and iN D4 media (NBM media + 1:50 B27 + BDNF, GDNF, CNTF (10 ng/mL, Peprotech) with 10 µM ROCK inhibitor and 2.0 μg/mL doxycycline. Cryopreserved day 4 stocks were thawed in iN media with 10 µM ROCK inhibitor and 2.0 μg/mL doxycycline and plated at 53 k cells/cm2. From day 4 to the end of differentiation/start of co-culture day 21, cells were cultured in iN media and fed every 3–4 days.

#### MSD ELISA

Samples were each diluted with 1% Blocker A (MSDR93BA-4) in wash buffer (Thermo TBS 25 mM TRIS, 0.15 M NaCl, pH 7.5 supplemented with 0.05% Tween). For our homebrew assay on the Meso Scale Discovery (MSD) electrochemiluminescence platform, each well of an uncoated 96-well multi-array plate (Meso Scale Discovery, #L15XA-3) was coated with 30 μL of a PBS solution containing capture antibody (3 μg/mL mouse 266, a monoclonal that binds to the mid-region of Aβ monomers [murine analog of the clinical antibody Solanezumab, for all human Aβ ELISAs) and incubated standing at RT overnight followed by blocking with 5% Blocker A in wash buffer for 90 min at RT with shaking at > 800 rpm. The coated plates were washed thrice with 150 μL of wash buffer, and 25 μL of diluted samples were added to the wells and incubated for 90 min shaking at 800 rpm. A detection antibody solution was prepared with 100 ng/mL biotinylated detection antibody 21F12 [12] and Streptavidin Sulfo-TAG (Meso Scale Discovery, #R32AD-5), diluted in wash buffer containing 1% Blocker A. The plate was washed thrice after sample incubation and 25 μL of the detector solution is added to each well and shaken for 90 min at RT. The wells were washed thrice and 100 μL of 2 × diluted MSD Read Buffer (MSD Read Buffer T (4x) R92TC-1) was added to each well and the plate was read and analyzed according to the manufacturer’s protocol on the MSD reader. The antibodies used to detect specific antigens were for human Aβ (x–42 specific).

#### SMCxPRO immunoassay

The SMCxPRO platform (Sigma Millipore) is based on single-molecule-counting technology [30] and typically allows a 20–100-fold increase in sensitivity compared with traditional immunodetection systems. The neutralized sample (25 μL/well) was then transferred to a black 384-well-read plate (Aurora) and read by the SMCxPRO instrument. Samples are detected with fluorescently labeled antibodies and the plate reader excites each well with a 642 nm laser that passes through the interrogation space. The resulting emitted light is measured using a confocal microscope lens and a photon detector. The output from the detector is a train of pulses, with each pulse representing one photon that was detected. The lower limit of reliable quantification (LLoQ) was defined as the lowest back interpolated standard that provides a signal two-fold the background with a percentage of recovery calculated between 80 and 100% and coefficient of variance (CV) ≤ 20%.

#### Immunohistochemistry: immunofluorescent staining

For free-floating Aβ peptide-specific stains, mouse brain sections were treated with 88% formic acid for 15 min for epitope retrieval and then washed thrice in PBS containing 0.3% Triton-X100 (PBSX). Samples were then blocked for 1 h in 5% normal donkey serum (Abcam) diluted in PBSX to reduce non-specific antibody binding followed by overnight incubation with primary antibodies (71A1 2 μg/mL) in blocking solution at 4 °C overnight. After 3 washes with PBSX, sections were then incubated with Alexa647-labeled donkey anti-mouse secondary antibodies (Biolegend) diluted 1000 × in 1% donkey serum in PBSX for 1 h. Samples were then washed thrice in PBSX and stained with DAPI (abcam 10 mM) diluted 5000 × in PBSX for 10 min. Stained sections were directly mounted on MAS-GP™ Adhesion Microscope Slides (Matsunami) and stored at 4 °C before fluorescent imaging.

#### Quantification and statistical analysis

All statistical analysis was carried out using GraphPad Prism 10 software. Statistical details of experiments are described in the text and figure legends.

## Results

### 71A1 Ab binding and oligomer vs. monomer selectivity

Surface plasmon resonance** (**SPR) in kinetic mode (see Methods) was used to evaluate the affinity and selectivity of 71A1 towards synthetic Aβ40 monomers vs. disulfide crosslinked Aβ40 dimers. Antibodies were covalently immobilized on the sensor surface. Lecanemab (to wt Aβ) and donanemab (specific to pGlu-3 Aβ) were used as positive and negative control antibodies, respectively, while anti-human IgG served as a technical negative control. Both 71A1 and lecanemab bound to Aβ40(S26C)_2_ dimers with high affinities (K_D_ 45.9 ± 34.5 nM for 71A1 and K_D_ 1.4 ± 0.02 nM for lecanemab), and both displayed no detectable binding to Aβ40 monomers, indicating their selectivity for aggregated forms of Aβ (Table 1). 71A1 exhibited a fast association rate (k_a_ = 1.99 × 10^5^ M^−1^ s^−1^), though slower than lecanemab (k_a_ = 2.28 ± 4.66 × 10^6^ M^−1^ s^−1^). Both antibodies showed relatively slow dissociation rates (k_d_ = 9.15 ± 5.63 × 10^–3^ s^−1^ for 71A1 and k_d_ = 3.25 ± 0.0201 × 10^–3^ s^−1^ for lecanemab). As expected, the negative control antibody donanemab, which only binds to a N-terminal pyroglutamate form of Aβ (pE3-42) [31, 32], did not show binding to either Aβ40(S26C)_2_ or Aβ40 monomers used in this experiment. Collectively, the SPR data show the selectivity of 71A1 towards Aβ oligomers over monomers and illustrate 71A1’s immunoreactive properties.

**Table 1:**
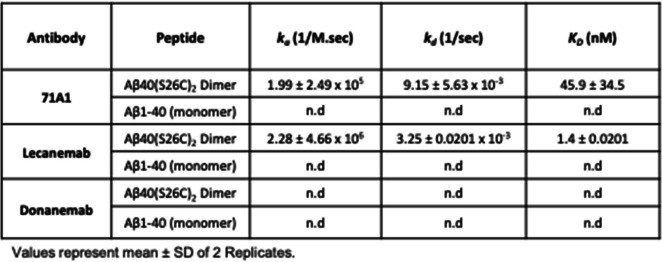
SPR binding kinetics of antibodies to synthetic Aβ peptides. Surface plasmon resonance (SPR) analysis was used to determine the affinity of antibodies 71A1, lecanemab, and donanemab to both Aβ1-40 monomers and Aβ1-40(S26C)_2_ covalent dimers. Kinetic rate constants k_a_ and k_d_ as well as affinity (K_D_), were measured using an Affinité Instrument at 25°C. Antibodies were immobilized on amino-coated sensors to assess interactions with both Aβ40 monomers and covalent Aβ40 dimers. Values are means ± SD of 2 replicates. n.d = not detected

### 71A1 immunopurifies neuroactive Aβ species from aqueous extracts of AD brain

**Fig. 1:**
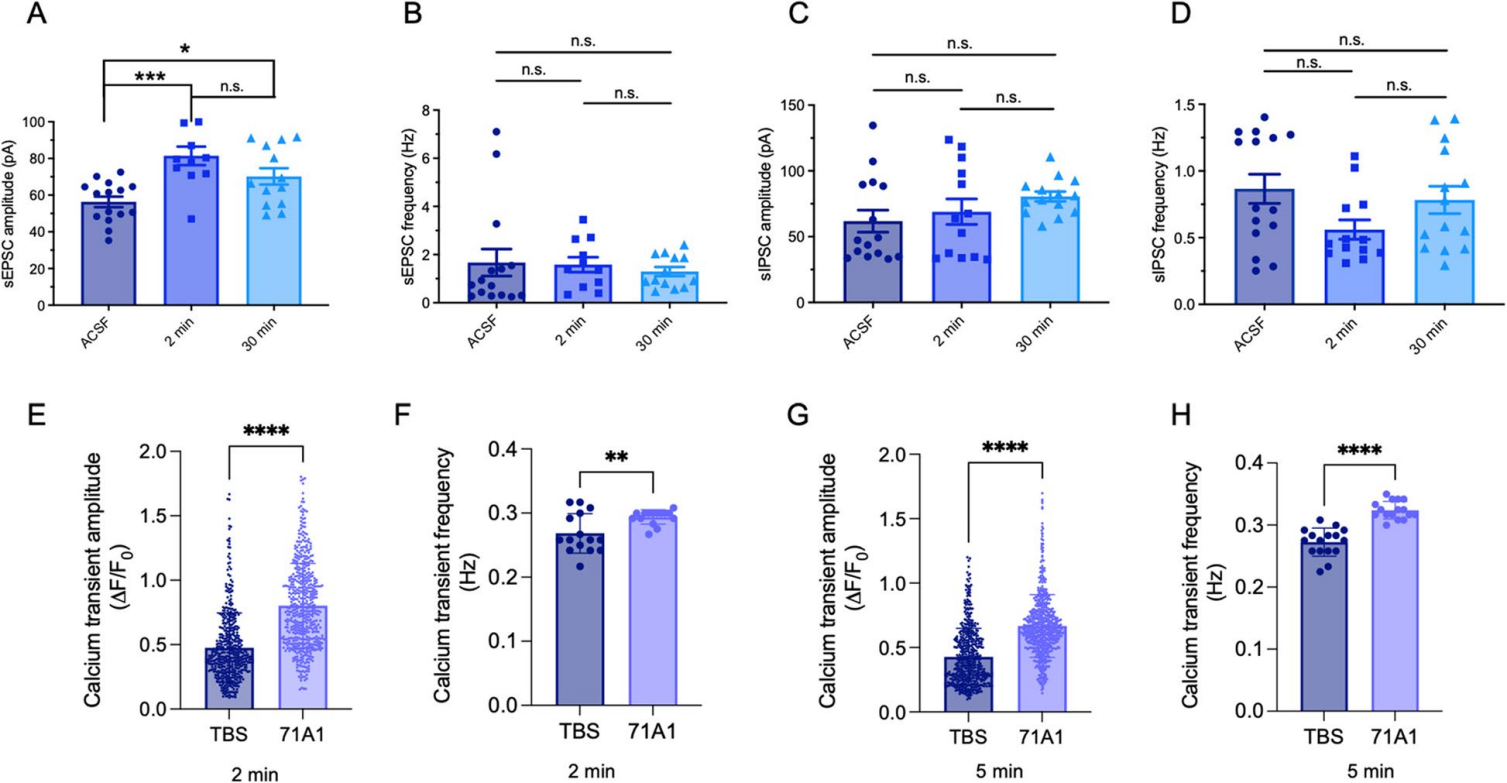
Electrophysiological analysis of bioactive 71A1-reactive Aβ species purified from AD brain. 71A1-reactive Aβ species impact excitatory synaptic signaling and calcium dynamics in rat primary neurons. **A**, **B** Changes in amplitude and frequency of spontaneous excitatory postsynaptic currents (sEPSCs) in DIV16-19 wt rat hippocampal neurons treated with aCSF (*n* = 15) or 71A1-affinity purified oAβ for 2 min (*n* = 11) and 30 min (*n* = 13). **C**, **D** Changes in amplitude and frequency of spontaneous inhibitory postsynaptic currents (sIPSCs) in neurons treated with aCSF (*n* = 15) or 71A1-purified oAβ for 2 min (*n* = 13) and 30 min (*n* = 14). **E**–**H** Calcium transient amplitude and frequency in jRGECO1a-expressing rat primary cortical neurons treated with TBS (control) or 71A1-purified Aβ at 2 min (E, *n* = 483, *n* = 531; F, *n* = 15; events across 15 individual cells) (**E**, **F**) and 5 min (G, *n* = 522, *n* = 625; H, *n* = 16; events across 16 individual cells) (**G**, **H**). * *p* < 0.05, ** *p* < 0.01, **** *p* < 0.001

In our initial oAβ assay study [17], we observed that 71A1 could protect against Aβ-induced synaptotoxicity of aqueous AD brain extracts. Here, we conducted additional experiments treating rat primary hippocampal neurons (DIV 16–19) with 71A1 affinity-purified oAβ from AD brain soaking extracts [22]. We initially assessed neuronal excitability by measuring spontaneous excitatory and inhibitory currents (sEPSC and sIPSC) as well as calcium transients. 71A1-immunoprecipitated human oAβ species increased hippocampal neuronal excitability. Specifically, whole-cell patch clamp recordings of pyramidal glutamatergic neurons showed that 71A1-purified oAβ significantly elevated sEPSC amplitude (Fig. 1A) but did not change sEPSC frequency (Fig. 1B). Compared to control treatment with aCSF (56.35 ± 2.89 pA, *n* = 15), a 2-min treatment with 71A1-purified oAβ resulted in a significant increase in the sEPSC amplitude (81.42 ± 5.05 pA, *n* = 11, *p* < 0.001). A longer, 30-min treatment also led to an increase in sEPSC amplitude (70.25 ± 4.48 pA, *n* = 13, *p* < 0.05). There was no significant change in sEPSC frequency among these 3 treatment conditions: aCSF control (1.67 ± 0.56 Hz, *n* = 15), 2-min treatment (1.58 ± 0.31 Hz, *n* = 11), and 30-min treatment (1.30 ± 0.19 Hz, *n* = 13).

In analogous recordings of sIPSC inhibitory currents, neither frequency nor amplitude were affected by exposure to 71A1-purified oAβ (Fig. 1C and D). The amplitudes of these 3 treatment conditions were aCSF control (61.83 ± 8.34 pA, *n* = 15), 2-min treatment (68.97 ± 9.71 pA, *n* = 13), and 30-min treatment (80.52 ± 3.73 pA, *n* = 14), and the frequencies were aCSF control (0.87 ± 0.11 Hz, *n* = 15), 2-min treatment (0.56 ± 0.07 Hz, *n* = 13), and 30-min treatment (0.78 ± 0.10 Hz, *n* = 14), respectively.

We next assessed the effects of 71A1-enriched oAβ on calcium transients in DIV16-19 rat cortical neurons expressing jRGECO1a, a genetically encoded calcium sensor under the synapsin promoter [33]. We observed that 71A1-purified oAβ increased both the amplitude (Fig. 1E and G) and frequency (Fig. 1F and H) of calcium transients following 2 or 5 min of oAβ exposure. After 2 min, there was a significant difference (*p* < 0.0001) in the average amplitude between neurons treated with TBS (0.48 ± 0.01, *n* = 483) vs. with 71A1-purified oAβ (0.80 ± 0.01, *n* = 531). Similarly, there was a significant difference (*p* = 0.0054) in the calcium transient frequency between cells treated with TBS (0.27 ± 0.01, *n* = 15) vs. with 71A1-purified oAβ (0.29 ± 0.003, *n* = 15). This was observed again after a 5-min exposure, showing a significant difference (*p* < 0.0001) in amplitude between cells treated with TBS (0.43 ± 0.01, *n* = 522) and vs. with 71A1-purified oAβ (0.67 ± 0.01, *n* = 625). Additionally, there was a significant difference (*p* < 0.0001) in calcium transient frequency between cells treated with TBS (0.27 ± 0.01, *n* = 16) and those treated with 71A1-purified oAβ (0.32 ± 0.004, *n* = 16). We conclude that diffusible oAβ isolated from AD brain (without plaque homogenization) using the 71A1 antibody potently induces abnormal neuronal hyperactivity.

### Development and optimization of an oAβ-preferring plate-based assay

**Fig. 2:**
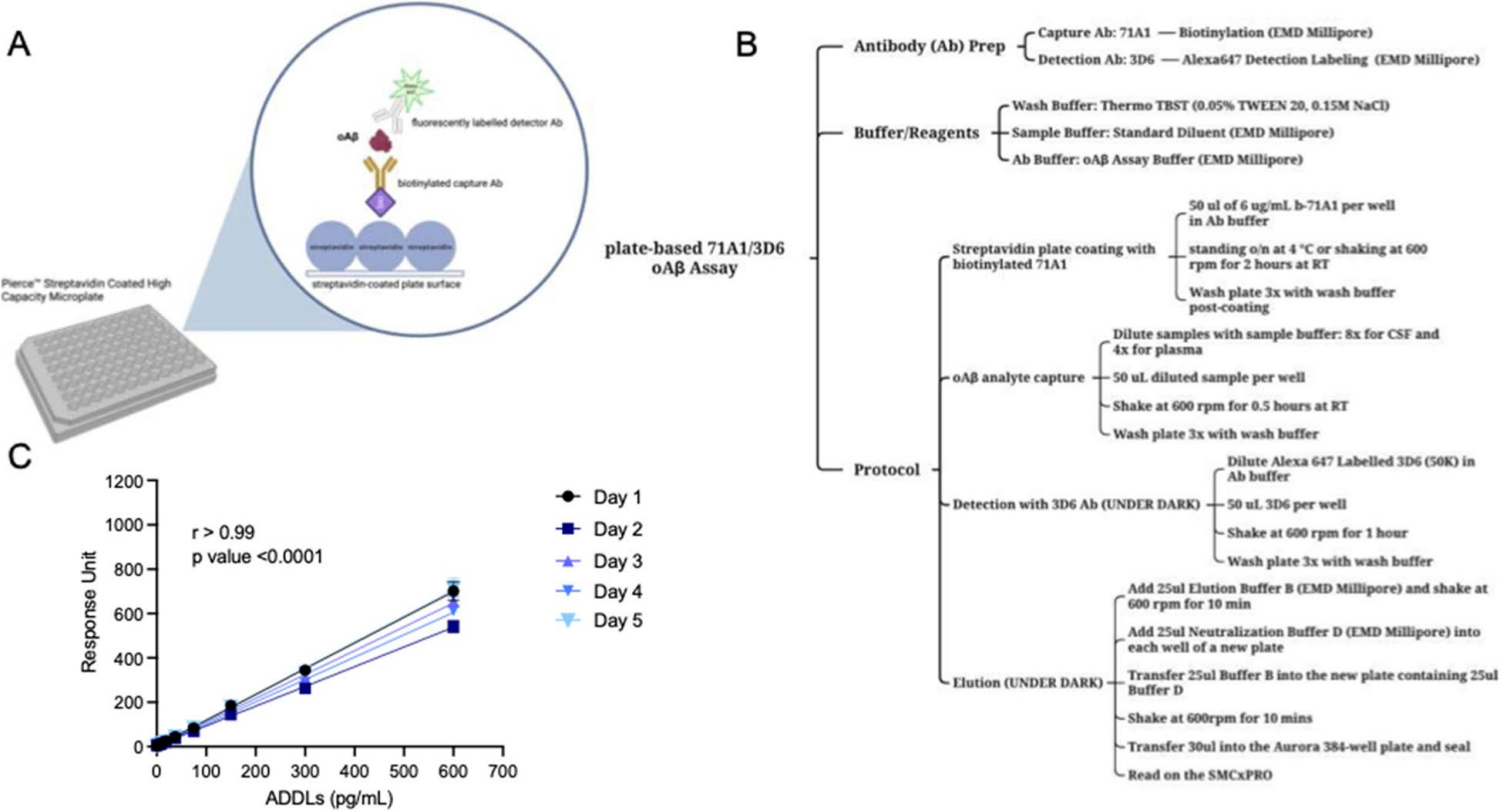
New plate-based assay platform and optimized protocol for oAβ detection. **A** Schematic of the updated plate-based sandwich immunoassay with biotinylated 71A1 capture antibody and alexa647-labelled 3D6 detector antibody. **B** Step-by-step overview of the assay protocol, detailing incubation conditions and workflow specific to the new plate-based format. **C** Consistency of calibrator curves: readouts of 71A1/3D6 oAβ assay using two-fold dilution series from 600 pg/mL using synthetic Aβ-derived diffusible ligands (ADDLs) as calibrator across five independent daily runs. Points on the graph represent the relative response units produced at each concentration of ADDLs

We performed multiple troubleshooting experiments to modify and optimize our prior bead-based immunoassay to quantify more effectively the 71A1-reactive oAβ species in CSF and plasma. Drawing from methods established in prior work, namely our 1C22/3D6 and 71A1/3D6 oAβ sandwich ELISAs on EMD Millipore’s Singulex Erenna platform [17, 34], we decided to continue using the 71A1/3D6 antibody combination to quantify the relatively low MW bioactive Aβ oligomers [17] that are present in human CSF and plasma.The assay was transferred from a magnetic Dynabead-based protocol to a plate-based platform using Streptavidin Coated High-Capacity Plates (Thermo Scientific™ #15,503) for the ELISA. Biotinylated capture antibody 71A1 was immobilized onto streptavidin-coated plates for analyte binding, followed by detection with the Asp-1 specific 3D6 (labeled with Alexa-647 dye). The resulting fluorescence was quantified by a SMCxPRO immunoassay plate reader (a newer version of the Singulex Erenna platform) (Fig. 2A).

We performed checkerboard titration experiments to optimize various parameters to improve assay performance, including screening a range of capture and detector antibody concentrations, incubation times, and reaction volumes. This comprehensive fine-tuning led to a considerably more operator-friendly workflow (Fig. 2B) and ensured more consistent analyte measurements (detailed below).

We constructed the oAβ sandwich ELISA with streptavidin-coated microplates as follows. All incubations were conducted at room temperature (RT, 25 °C). Plates were shaken at 600 RPM on either a microplate shaker (Quanterix) or a Jitterbug shaker (Boekel) in the dark to avoid light-sensitive conditions. Wells of Streptavidin Coated High-Capacity Microplates (Pierce) were coated with 50 μL of a 6 μg/mL solution of biotinylated 71A1 in Oligomer Aβ Assay Buffer (see Methods) (proprietary, made by EMD Millipore 02–0607-00 specifically for the 71A1/3D6 assay). The plate was incubated either undisturbed overnight at 4 °C or shaken at 600 rpm for 2 h at RT followed by washing 3 × in TBS-T (Pierce™ 28,360; 25 mM Tris, 0.15 M NaCl, 0.05% Tween™−20) with a Biotek 405 TS washer. Then, all samples and ADDL oAβ standards were diluted in a 0.22 μm-filtered solution of Standard Diluent (EMD Millipore 02–0485-00). 50 μL of the sample or ADDL standard diluted to a specified concentration were added to each well and incubated 0.5 h on a plate shaker at 600 rpm. To establish the lower limit of reliable quantification (LLoQ), we generated a 12-point standard curve using synthetic Aβ42 ADDLs [1] at concentrations from 0 to 600 pg/mL, each assayed in duplicate. The resultant ADDL standard curve was used to normalize the relative fluorescence response units of the biological samples to calculate analyte concentrations (in pg/mL). Biological samples such as aqueous AD brain extracts, CSF, and plasma were diluted in Standard Diluent and loaded in triplicate. Following the 0.5 h sample incubation, wells were washed thrice with TBS-T, and 50 μL of 3D6-Alexa 647 conjugate diluted at 50,000x (~ 100 ng/mL) in Oligomer Aβ Assay Buffer were added to the wells and incubated with shaking for 1 h in the dark to prevent photobleaching of the Alexa 647 dye. Wells were then washed 3 times with TBS-T.

To elute the detection antibody, 25 μL of a 0.1 M glycine solution (pH 2.7) containing 0.01% Triton X-100 (Buffer B from EMD Millipore) were added to each well and incubated under dark on a plate shaker for 15 min. The 25 μL eluent solutions were then transferred to a new 96-well plate (Axygen V-bottom), where each well contained 25 μL of neutralization buffer (Buffer D from EMD Millipore). The 50 μL of neutralized eluent was shaken for 15 min under dark. 25 μL of this mixture was transferred to the wells of a 384-well plate (Aurora ABB2-00160A) and the fluorescence measured using an SMCxPRO plate reader. We determined the lower limit of detection (LLoD) and the LLoQ based on the fluorescent response signal directly obtained from the instrument, calculated using Eqs. 1 and 2:

1 $$\mathrm{LLoD}=2\times s\mathrm{tandard}\;\mathrm{deviation}\;\mathrm{of}\;\mathrm{response}\;\mathrm{of}\;\mathrm{background}\;/\mathrm{slope}\;\mathrm{of}\;\mathrm{the}\;s\mathrm{tandard}\;\mathrm{curve}.$$

2 $$\mathrm{LLoQ}=\;\mathrm t\mathrm h\mathrm e\;\mathrm l\mathrm o\mathrm w\mathrm e\mathrm s\mathrm t\;\mathrm b\mathrm a\mathrm c\mathrm k-\mathrm i\mathrm n\mathrm t\mathrm e\mathrm r\mathrm p\mathrm o\mathrm l\mathrm a\mathrm t\mathrm e\mathrm d\;\mathrm c\mathrm o\mathrm n\mathrm c\mathrm e\mathrm n\mathrm t\mathrm r\mathrm a\mathrm t\mathrm i\mathrm o\mathrm n\;\mathrm o\mathrm n\;\mathrm t\mathrm h\mathrm e\;\mathrm s\mathrm t\mathrm a\mathrm n\mathrm d\mathrm a\mathrm r\mathrm d\;\mathrm c\mathrm u\mathrm r\mathrm v\mathrm e\;\mathrm w\mathrm i\mathrm{th}\;\mathrm a\mathrm n\mathrm a\mathrm l\mathrm y\mathrm t\mathrm e\;\mathrm r\mathrm e\mathrm c\mathrm o\mathrm v\mathrm e\mathrm r\mathrm y\;\mathrm o\mathrm f\;100\pm20\%,\;\mathrm a\;\mathrm r\mathrm e\mathrm s\mathrm p\mathrm o\mathrm n\mathrm s\mathrm e\;2x\;\mathrm{greater}\;\mathrm{than}\;\mathrm{background}\;\mathrm{signal},\;\mathrm a\mathrm n\mathrm d\;\mathrm a\;\mathrm{co}\mathrm e\mathrm f\mathrm f\mathrm i\mathrm c\mathrm i\mathrm e\mathrm n\mathrm t\;\mathrm o\mathrm f\;\mathrm v\mathrm a\mathrm r\mathrm i\mathrm a\mathrm n\mathrm c\mathrm e\;(\mathrm{CV})\;\mathrm o\mathrm f\;\leq<span class='crossLinkCiteEqu'>20</span>\%.\;\mathrm T\mathrm h\mathrm i\mathrm s\;\mathrm c\mathrm a\mathrm l\mathrm c\mathrm u\mathrm l\mathrm a\mathrm t\mathrm i\mathrm o\mathrm n\;\mathrm i\mathrm s\;\mathrm b\mathrm a\mathrm s\mathrm e\mathrm d\;\mathrm o\mathrm n\;\mathrm t\mathrm h\mathrm e\;\mathrm r\mathrm{es}\mathrm p\mathrm o\mathrm n\mathrm s\mathrm e\;\mathrm s\mathrm i\mathrm g\mathrm n\mathrm a\mathrm l\;\mathrm o\mathrm f\;\mathrm t\mathrm h\mathrm e\;\mathrm s\mathrm y\mathrm n\mathrm t\mathrm h\mathrm e\mathrm t\mathrm i\mathrm c\;\mathrm s\mathrm t\mathrm a\mathrm n\mathrm d\mathrm a\mathrm r\mathrm d\;\mathrm c\mathrm a\mathrm l\mathrm i\mathrm b\mathrm r\mathrm a\mathrm t\mathrm o\mathrm r\mathrm s\;\mathrm r\mathrm u\mathrm n\;\mathrm o\mathrm n\;\mathrm e\mathrm a\mathrm c\mathrm h\;\mathrm p\mathrm l\mathrm a\mathrm t\mathrm e.$$

Figure 2C shows ADDL standards ranging from 0 to 600 pg/mL measured with the plate-based assay on 5 days to show reproducibility and sensitivity. Across 5 runs on different days, the standard curves exhibited minimal variation in slope (< 6.7%), yielding an average LLoQ of 2.5 pg/mL. This detection range left ample room to cover the entire spectrum of detectable analyte concentrations at appropriate dilutions of both human CSF and plasma (see below).

### Selectivity of the oAβ assay

In prior work [17], we showed that 71A1 & 3D6 antibodies can form a sandwich assay that specifically detects Aβ oligomers. The transition of the assay from a bead-based to a plate-based format was prompted by issues we encountered with inconsistent quality among production lots of beads, some of which exhibited non-specific binding in both plasma and aqueous brain homogenates, leading to unstable measurements and sometimes high CVs.

**Fig. 3:**
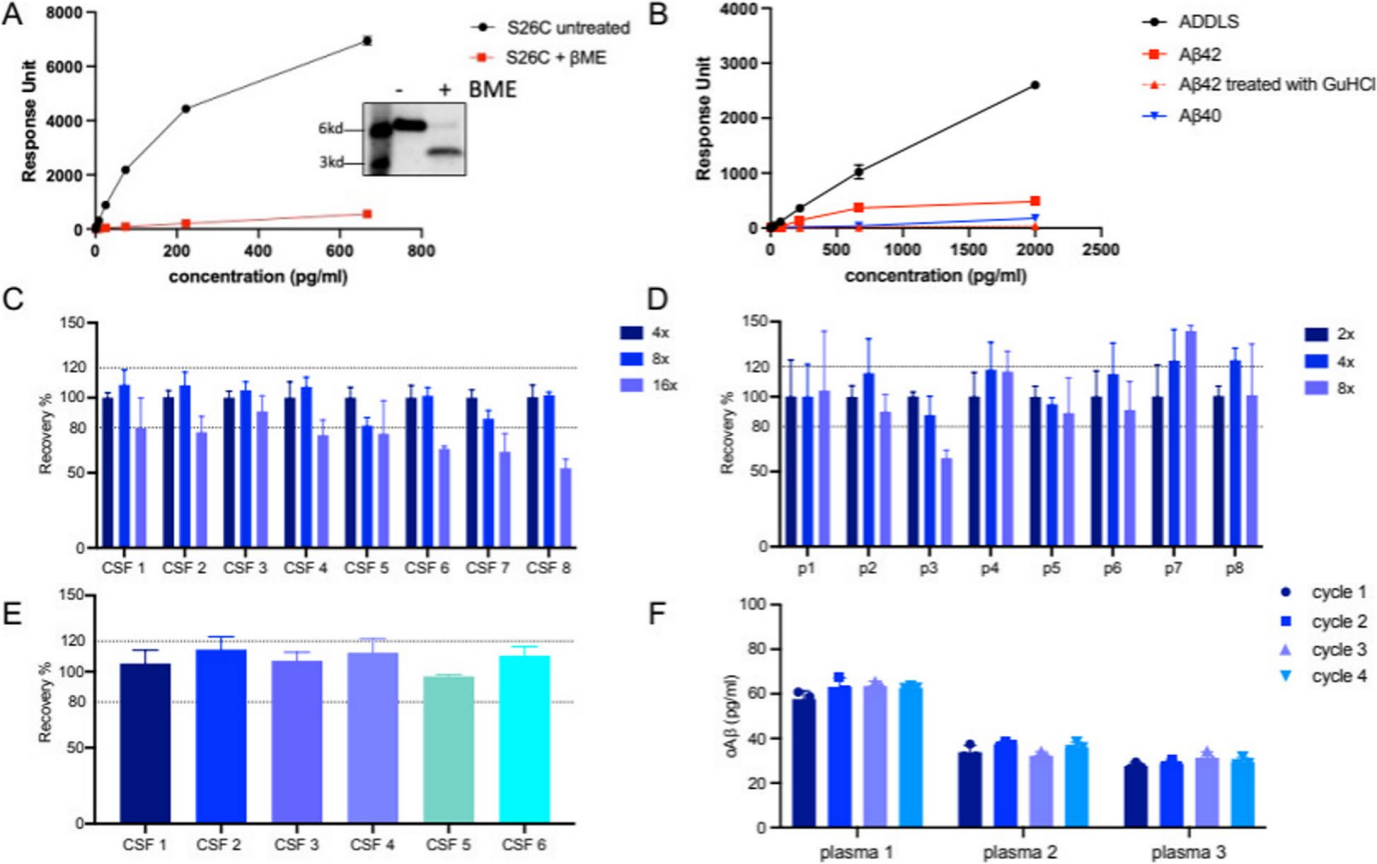
Validation of signal specificity of the plate-based 71A1/3D6 oAβ immunoassay. **A** Specific detection by the 71A1/3D6 assay of synthetic (S26C)_2_ Aβ1-40 disulfide-crosslinked dimers (black) vs. the depolymerized Aβ1-40 monomers post-βME reduction (red). Inset: SDS-PAGE WB of these samples. **B** Specificity of the 71A1/3D6 assay for synthetic Aβ-derived diffusible ligands (ADDLs, in black) vs. synthetic Aβ1-40 monomers (blue); and synthetic Aβ1-42 monomers (red) before (red squares) and after (red triangles) depolymerization in 7 M GuHCl. **C** Signal recovery (percentages) of 8 individual human cerebrospinal fluids (CSF) measured at 4x, 8x, or 16 × dilutions by the 71A1/3D6 assay, with results normalized to 4 × dilution. **D** Signal recovery (percentages) of 8 individual human plasmas measured at 2x, 4x, and 8 × dilutions by the 71A1/3D6 assay, with results normalized to 2 × dilution. **E** Signal recovery (percentages) in the 71A1/3D6 assay by detecting 500 pg/mL of ADDLs spiked into 6 individual human CSF samples. **F** 71A1/3D6 oAβ signal stability in three individual human plasmas over four freeze–thaw cycles

To avoid this problem, we examined potentially confounding variables that could lead to non-specific binding of biological samples to the streptavidin plate alone (no capture antibody). We detected no binding of either plasma or brain homogenate in the absence of the immobilized 71A1 (data not shown). This negative result suggests that the signal observed in the 71A1/3D6 sandwich assay originates from 71A1-immunoreactive species, presumably Aβ oligomers rather than monomers or background. Two lines of evidence support this statement. First, using synthetic Aβ40(S26C)_2_ disulfide crosslinked dimers [35], a major reduction (> 95% decrease) of the 71A1/3D6 signal was seen when the reducing agent 2-Mercaptoethanol (βME) was added to reduce the dimers to monomers (Fig. 3A). To equalize any effects βME may have had on the readout, all results comparing the protein calibrators (ADDLs) to βME-treated Aβ40(S26C)_2_ were generated by spiking equivalent amounts of βME into the ADDLs solution. Second, the cross-reactivity of the oAβ assay with Aβ monomers was further assessed using solutions of synthetic Aβ1–40 monomers and of Aβ1-42 monomers with and without the addition of the Aβ oligomer-depolymerizing denaturant GuHCl (guanidinium hydrochloride) (Fig. 3B). The synthetic Aβ1–40 and Aβ1–42 monomers had been purified by size exclusion chromatography (SEC) to ensure only monomeric forms of the protein were present at the time of aliquoting and storage at −80 °C. Compared with Aβ1–40, the more hydrophobic Aβ1–42 was more prone to aggregation, as expected, and needed to be dissociated into monomers with 5 M GuHCl (which was then diluted) before measuring with the oAβ assay. The percent cross-reactivity was calculated by dividing the observed ELISA values by the expected values based on an ADDLs standard curve at comparable protein concentrations. The calculated percent cross-reactivity of monomers was 17% at 2,000 pg/mL of Aβ42 peptide before GuHCl but fell to a negligible 0.25% after GuHCl depolymerization into monomers. For the Aβ40 monomer at 2,000 pg/mL, it was 5%. Synthetic Aβ monomers were assayed at these very high concentrations to mimic the high endogenous Aβ40 monomer levels present in human CSF. Thus, even at these very high monomer concentrations, the oAβ assay demonstrated markedly higher selectivity for aggregated than monomeric Aβ42 and monomeric Aβ40. Collectively, these findings indicate that our 71A1/3D6 plate assay consistently quantifies levels of oligomeric Aβ with high selectivity over Aβ monomers, which produce minimal non-specific signals (Fig. 3A, B).

### Validation of the new assay on endogenous human oAβ

After verifying the sensitivity, reproducibility, and specificity of our plate-based oAβ assay using synthetic ADDLs, we evaluated the assay’s ability to measure endogenous Aβ oligomers in human CSF and plasma. Initially, we tested CSF samples of 8 individuals from the BWH Memory and Aging Cohort (MAC) who had undergone evaluation in our Cognitive Disorders Division (Fig. 3C) (see Supplementary Table 1 for cohort demographics). The frozen CSFs were thawed and subjected to twofold serial dilutions from 4 × to 16x, and their oAβ levels were quantified, with results corrected for dilution factor. At 4 × and 8 × dilutions, signals from all 8 CSF samples exhibited close to 100% analyte recovery (Fig. 3C), with all signals falling in the 100% ± 20% range. At 16 × dilution, 7 out of 8 measured CSFs showed signals outside of the acceptable range of 80–120% recovery (Fig. 3C). Based on these results, we selected the 8 × dilution for future CSF assays as it requires the least amount of biological sample while maintaining very good signal recovery.

Next, we conducted the same twofold serial dilution recovery experiment on 8 individual plasmas from 8 different MAC cohort individuals (Fig. 3D). Due to lower oAβ levels present in plasma than CSF, experiments in plasma were performed using dilutions from 2 × to 8x. At an 8 × dilution, 2 out of 8 plasmas had signal recovery outside the 80–120% range (plasmas 3 and 7); at a 4 × dilution, 2 out of 8 were at the upper limit of the target recovery range (plasmas 7, 8). The rest of the plasmas were well within the target recovery range of 80–120%. The assayed plasmas were in the lower range of our ADDL standard curve and just above the LLoQ when tested at 8 × dilutions. To maintain good analyte recovery, a dilution factor is also chosen based on whether the diluted signal falls within the linear range of the standard curve for accurate back-interpolation of analyte concentrations. Based on the response signals and recovery results in Fig. 3C and D and concerns about any potential extraneous effects of the matrix of biological fluids, we chose 4 × dilution for plasma and 8 × for CSF as in an accurate response range for concentration calculations.

To further confirm that the signal measured by the assay is contributed almost exclusively by oligomers of Aβ, we conducted spike-and-recovery assays. We spiked in synthetic ADDLs into the undiluted CSFs of 6 individuals. After spiking, the CSF samples were assayed at their usual 8 × dilutions to reach a final concentration of 500 pg/mL ADDLs spiked in after dilution. The oAβ levels were then measured, and the analyte recovery was calculated using Eq. 3 [36]:

3 $$\%\;\mathrm{recovery}\;\mathrm{of}\;\mathrm{A\beta}=\left[\mathrm{oA\beta}\right]\;\mathrm{measured}\;\mathrm{in}\;\mathrm{CSF}\;\mathrm{after}\;\mathrm{ADDLs}\;\mathrm{spike}\;\mathrm{in}\;\left(\times100\right)\;/\mathrm{expected}\;\left[\mathrm{oA\beta}\right]\;\left(\mathrm{spiked}\;\mathrm{in}\;\mathrm{ADDLs}\;+\;\mathrm{endogenous}\;\mathrm{oA\beta}\;\mathrm{in}\;\mathrm{CSF}\right)$$

The mean and median endogenous oAβ value of the 6 CSFs was 652.9 pg/mL and 271.9 pg/mL respectively (8 × diluted). The results revealed that the recovery of the 6 individual CSF samples ranged from 97 to 115% (105.4 ± 8.2%, 114.5 ± 7.4%, 107.1 ± 5.3%, 112.4 ± 8.1%, 96.9 ± 1.1%, 110.4 ± 5.5% respectively) (Fig. 3E). These results indicate that the new plate-based assay retains its ability to accurately detect a specific target signal in the complex microenvironment of human biofluids. The serial dilution and spike-and-recovery testing collectively suggest that the plate-based oAβ assay remains precise and sensitive when assaying human biofluids.

To ensure the consistency and reliability of the oAβ assay when analyzing clinical samples across different assay runs and on different days, we calculated the CV% for a single pooled plasma sample (from 10–15 humans) analyzed on 4 different plates on the same day, as well as the CV% of 4 plates analyzed on each of 10 different days (Table 2). Intra-plate CV% for samples analyzed on the same day ranged between 5 and 14%. The average inter-day CV% across all 10 run days was 12%. The single pooled human plasma sample from 10–15 different individuals serves as our internal control sample for quality control (QC) and is run on every oAβ assay to ensure technical consistency.

**Table 2:**
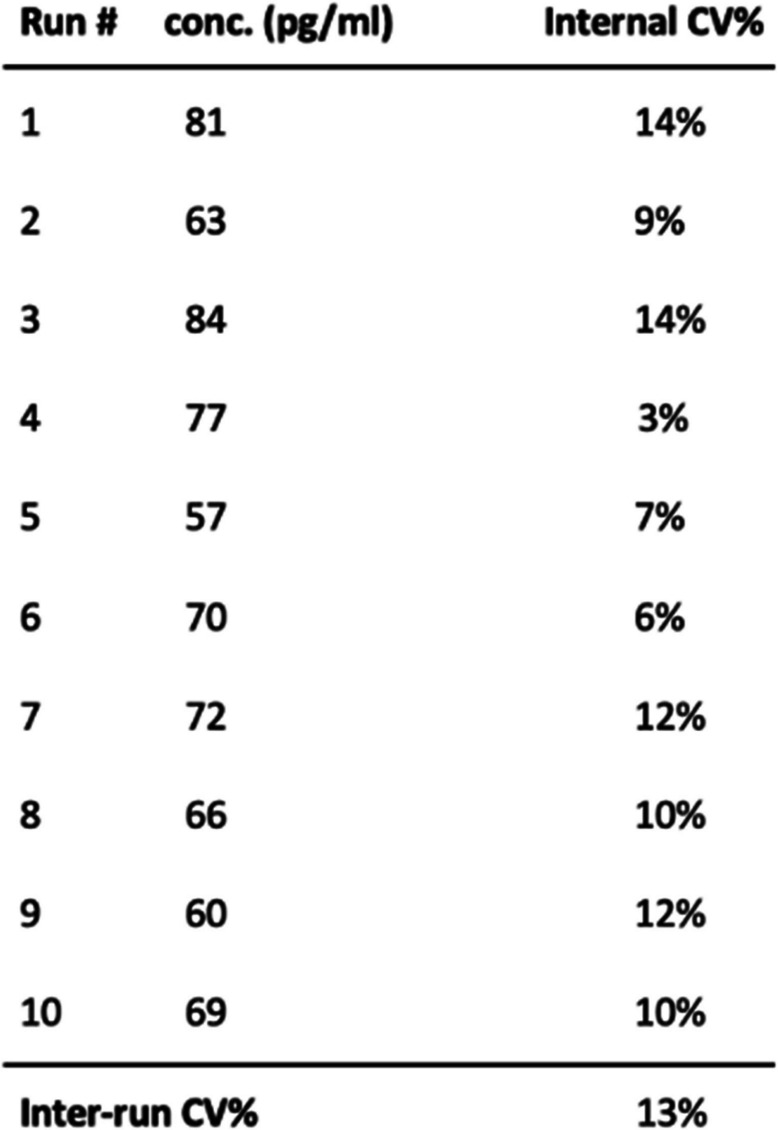
Assay performance evaluated by repeat testing of a single pooled human plasma sample. Internal variation was determined by calculating the coefficients of variation (CV%) of values measured for the same single pooled plasma sample run on 4 different assay plates in one day. Inter run variation was determined by calculating the coefficients of variation (CV%) of values measured for the same single pooled plasma sample run on 10 different days. This procedure was repeated on each of the 10 days and the CV% calculated across all 10 days

**Fig. 4:**
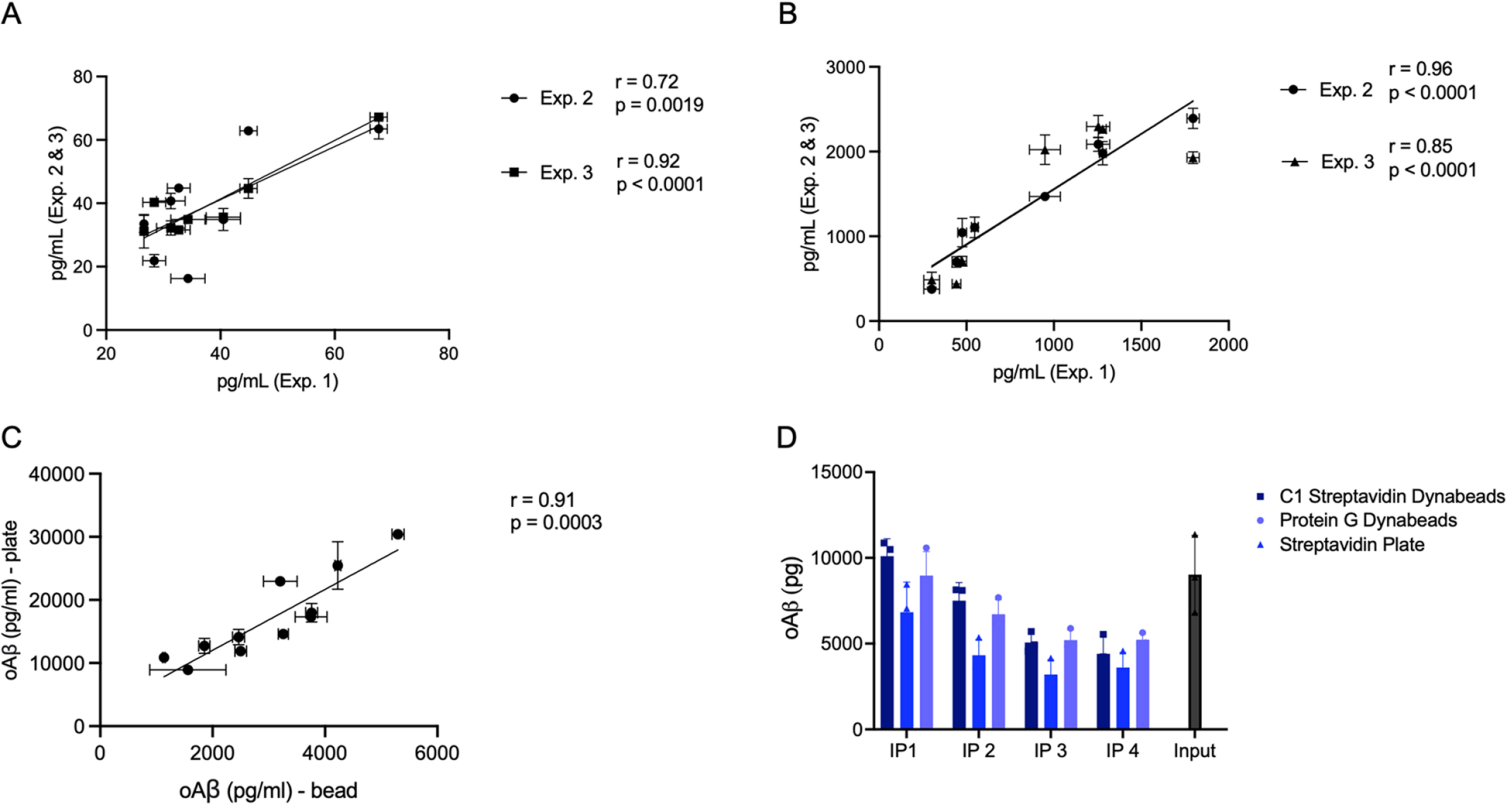
Validation of plate-based 71A1/3D6 oAβ assay consistency over time. Correlation of 71A1/3D6 oAβ readouts from 8 individual human plasmas (**A**) and 8 individual human CSFs (**B**) across three independent assay runs, with oAβ concentrations from experiment 1 on the x-axis and oAβ concentrations from experiments 2 and 3 on the y-axis. **C** Correlation of oAβ measurements of 11 individual human CSFs on the previous bead-based assay [17] versus on the new plate-based assay, demonstrating consistency between the assay platforms. **D** plate-based 71A1/3D6 oAβ measurement of post IP’ed supernatants from one human (AD) brain soaking extract immunodepleted with 71A1 conjugated to either C1 streptavidin Dynabeads or protein G Dynabeads or Streptavidin microplates

Next, we assessed the assay's consistency and reproducibility in measuring individual human plasma and CSF samples. As shown in Fig. 4A and B, the plate-based oAβ assay was applied to detect 8 individual plasmas (at 4 × dilution) and 8 individual CSFs (at 8 × dilution). These samples were measured in 3 independent experiments run on different days: the results from experiments 2 and 3 were then plotted against those from experiment 1, and linear regressions of their correlations were calculated. For the 8 individual plasmas, the correlation coefficients when runs 2 and 3 were plotted against run 1 were *r* = 0.72, *p* = 0.0019 and *r* = 0.92, *p* < 0.0001 respectively, supporting the assay’s ability to provide consistent measurements of the plasma oAβ analyte. Similarly, for the 8 individual CSFs, the correlation coefficients when runs 2 and 3 were plotted against run 1 were *r* = 0.96, *p* < 0.0001 and *r* = 0.85, *p* < 0.0001 respectively. The correlations between runs 2 and 3 were also significant in CSF and plasma (Supp. Figure 1). These data support the assay's reliability in detecting oAβ levels in individual biological samples assayed across multiple plates and experimental days.

### The effect of freeze–thaw cycles on measured levels of oAβ in plasma

Our previous observations on plasma samples from various subjects in earlier work indicated a potential decrease in monomeric Aβ detection following multiple freeze–thaw cycles (data not shown). Here, we assessed the effects of freeze–thaw cycles on oAβ concentration in biological fluids. Aliquots of 3 pooled plasma samples (each consolidated from 10–15 individuals) were subjected to 1 to 4 freeze–thaw cycles, and oAβ levels were measured using the plate-based assay. The oAβ levels remained stable across 4 freeze–thaw cycles (Fig. 3F). These results suggest that soluble oAβ species in plasma that are detected by the 71A1/3D6 assay exhibit a low tendency to be affected by sample storage and repeated use, at least up to 4 cycles.

### Binding efficacy of the new plate-based oAβ assay vs. the prior bead-based assay

We examined the relationship between oAβ levels detected by our earlier bead-based assay and new data obtained using the more stable and consistent plate-based assay. The plotted data (Fig. 4C) revealed a highly significant correlation (*p* = 0.0003) between these two platforms, with an *r*-value of 0.9091. Migrating a bead-based to a plate-based assay could present a considerable steric shift in the biochemical interactions involved in immobilizing a conformational antibody like 71A1 onto plate surfaces vs. onto free-floating beads in solution. To address whether plate-immobilized 71A1 retained binding properties and efficiency for oAβ, we immunoprecipitated (IP) 71A1-reactive molecules from aqueous soaking extracts of AD cortex [22] using 3 different pull-down methods to compare their efficacies (Fig. 4D). Native 71A1 or biotinylated 71A1 were immobilized respectively on either protein G magnetic dynabeads (Invitrogen #10003D) or C1 streptavidin-coated beads (Invitrogen #65,001) to represent the bead-based assay and compared with streptavidin-coated plates (Pierce #15,503) to provide the binding conditions of the new plate-based assay. To ensure that the analyte was pulled down to a high degree, we conducted four rounds of IP sequentially using the supernatants of each preceding round. We measured oAβ levels in both eluents (indicating the amount of oAβ pulled down by each IP) and supernatants (indicating the residual oAβ levels left). We observed no statistically significant differences among the three methods in the oAβ levels remaining in the post-IP supernatants after four sequential IP rounds (Fig. 4D). Maximal pulldown was achieved by the third IP round in each case, i.e. oAβ signals in the post-IP supernatants after the fourth round were the same as after the third round. The streptavidin-coated plate achieved oAβ pull-down comparable to both the protein G magnetic beads and the streptavidin-coated beads, indicating that immobilizing the conformational 71A1 antibody on a plate did not significantly decrease its oAβ binding efficiency.

### The assay detects changes in oAb that reflect Ab42 levels in the media of human neurons expressing APP and PS1 mutations

We used the new plate-based oAb assay to quantify the conditioned media (CM) from human induced pluripotent stem cells (iPSCs, DIV 21) harboring certain familial Alzheimer’s Disease (FAD) [26] mutations known to shift Aβ production. Compared to an isogenic control (1.07 ± 0.28 pg/μg), CM from neurons expressing the protective Icelandic A673T/A673T APP mutation contained significantly lower or undetectable oAβ levels (0 or below LLoQ). In contrast, cells with the APP^wt^/Swedish APP (APP^swe^) mutation exhibited a threefold increase in oAβ levels vs. control (3.3 ± 1.3 pg/μg) as expected, while CM from homozygous double-mutant neurons engineered to express APP^swe^ PSEN1^M146V^/APP^swe^ PSEN1^M146V^ showed the highest oAβ levels (8.9 ± 1.5 pg/μg) (S. Figure 2 A). These oAβ levels showed the same trends as Aβ42 monomer levels (post GuHCl depolymerization) measured in these CMs: 0.76 ± 0.29 pg/μg for the isogenic control, 0.48 ± 0.071 pg/μg for A673T/A673T, 1.1 ± 0.24 pg/μg for APP^swe^, and 3.2 ± 0.24 pg/μg for APP^swe^ PSEN1^M146V^/APP^swe^ PSEN1^M146V^ (S. Figure 2B). Interestingly, compared to the WT parental line, cells carrying the protective Icelandic mutation (A673T/A673T) showed a significant decrease in Aβ42 monomer levels, and their oAβ levels fell markedly to become undetectable. These results validate the assay’s ability to accurately reflect disease related endogenous Aβ42 overproduction and subsequent oligomer formation. This highlights the assay’s potential as both a diagnostic tool and a platform for screening and monitoring the responses to pharmacological modulators of presenilin/γ-secretase activity.

### Application of the assay to Ab oligomers in APP NL-G-F mouse brains

**Fig. 5:**
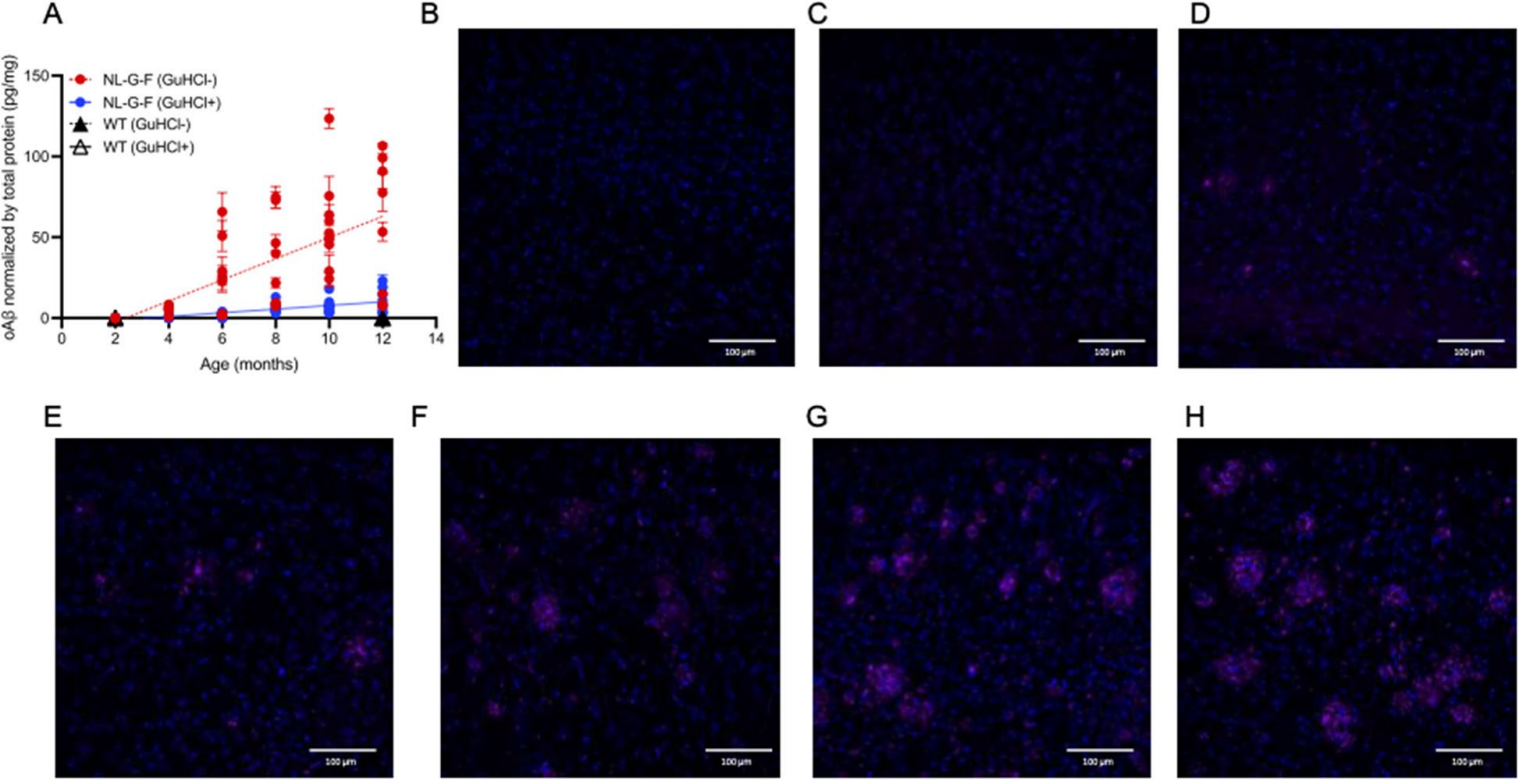
Longitudinal tracking of pathological Aβ change in aging NL-G-F mice with 71A1. **A** Plate-based 71A1/3D6 oAβ quantification of NL-G-F mouse brain homogenates at increasing ages (2 to 12 mo; *n* = 8–10 mice per age) before (red) and after (blue) depolymerization in 7 M GuHCl, confirming signal specificity for aggregated Aβ. WT mouse brain homogenates at 2 and 12 mo (*n* = 4 mice per age) were measured concurrently (black) as negative controls to highlight the absence of detectable oAβ, as expected (empty triangles (GuHCl +) overlap black triangles (GuHCl-) along x = 0). **B** Immunofluorescent staining by 71A1 of Aβ deposits from 12 mo WT mice. **C**-**H** Immunofluorescent staining by 71A1 of Aβ deposits in NL-G-F mouse hippocampal cryosections from 2 mo old (**C**) to 12 mo old (**H**) at 2 mo intervals, confirming an age-dependent rise in Aβ deposition

We next evaluated the utility of the plate-based assay for quantifying endogenous oligomeric Aβ species in aqueous brain extracts from the APP-NL-G-F knock-in mouse model that avoids heterologous overexpression of APP. We analyzed extracts from increasing ages of both sexes (5 females, 5 males) of APP NL-G-F mice at 2, 4, 6, 8, 10, and 12 mo, with each extract diluted 100-fold before assay. oAβ levels measured by the ADDL standard curve were normalized to total protein concentrations of the extracts (BCA assay) and reported as pg oAβ per mg of total protein (pg/mg). Results showed a clear age-dependent increase in brain oAβ. The signals were markedly reduced (< 10–20% remaining) following GuHCl denaturation, confirming the assay detects aggregated Aβ species (Fig. 5A).

In 16 wild-type mouse brain aqueous extracts (4 females and 4 males each at ages 2 and 12 mo), oAβ was undetectable (below LLoD), consistent with the Asp-1 3D6 detector antibody being human Aβ specific (Fig. 5A). In the human APP knock-in NL-G-F mouse brains, mean oAβ levels were below detection at 2 mo but increased with age: 5.6 ± 2.2 pg/mg at 4 mo; 22.7 ± 22.1 pg/mg at 6 mo; 32.2 ± 27.6 pg/mg at 8 mo; 57.3 ± 27.8 pg/mg at 10 mo; and 57.3 ± 42.1 pg/mg at 12 mo. Following GuHCl depolymerization, the oAβ signals fell markedly to: 0.1 ± 0.2 pg/mg at 4 mo; 1.5 ± 1.4 pg/mg at 6 mo; 5.6 ± 3.5 pg/mg at 8 mo; 8.0 ± 5.5 pg/mg at 10 mo; and 13.4 ± 11 pg/mg at 12 mo. These results confirm that the detected signal originates from aggregated Aβ species.

The age-dependent increase in oAβ levels in APP NL-G-F brain extracts supports the specificity for oligomeric species captured by the 71A1 antibody. We corroborated this age-related rise by immunohistochemistry (IHC) (Fig. 5B). Confocal microscopy revealed that 71A1 labeled extracellular Aβ deposits in PFA-fixed brain sections. Our previous studies [17] noted that Aβ staining by 71A1 in human brain tissue was diminished following PFA fixation, presumably due to its conformational epitope. This was also observed in the NL-G-F mouse brain slices; however, pretreatment of the fixed brain sections with 88% formic acid, a known antigen-retrieval method, before staining markedly improved the Aβ signal, even in PFA-fixed sections from both human (AD) and mouse brains, in accord with published findings [37]. We evaluated 71A1 labeling of Aβ deposits from age 2 to 12 mo (5 females and 5 males at each age) to match the samples prepared for aqueous extraction (Fig. 5). Diffuse plaques were observed as early as 4 mo, with a clear trend of increasing 71A1-reactive Aβ deposits with age. These age-dependent increases suggest that 71A1 effectively captures a dynamic rise in oAβ pathology both by immunoassay and IHC. To confirm 71A1 specificity, staining was also conducted on brains of age-matched wild-type C57/BL6 mice (2 and 12 mo), yielding no detectable signal (not shown).

### Clinical application to human Aβ oligomers in CSF and plasma

After extensively validating the new assay biochemically and on oAβ-promoting FAD human neurons and FAD APP knock-in mice, we began investigating its potential clinical utility by analyzing oAβ levels in human CSF. We used a cohort of 108 patients with varied cognitive diagnoses from the Memory and Aging Clinic (MAC) at Brigham and Women’s Hospital (see Supp. Table 1 for demographic and diagnostic information). All patients in this cohort presented with cognitive symptoms, and their CSF was collected and sent to Mayo Clinic Laboratories for biomarker measurements of Aβ42, p-Tau181, and total-tau (t-Tau)) on the ADEVL platform. AD diagnoses were based on the CSF biomarker level cutoffs from Mayo Clinic Labs as follows:

- Aβeta42): ≤ 834 pg/mL
- Total-Tau: > 238 pg/mL
- Phosphorylated-Tau 181: > 21.6 pg/mL
- *p*-Tau/Aβ42: > 0.028
- The > 0.028 cutoff for p-Tau/Aβ42 served as the determinant of a diagnosis of AD.

**Fig. 6:**
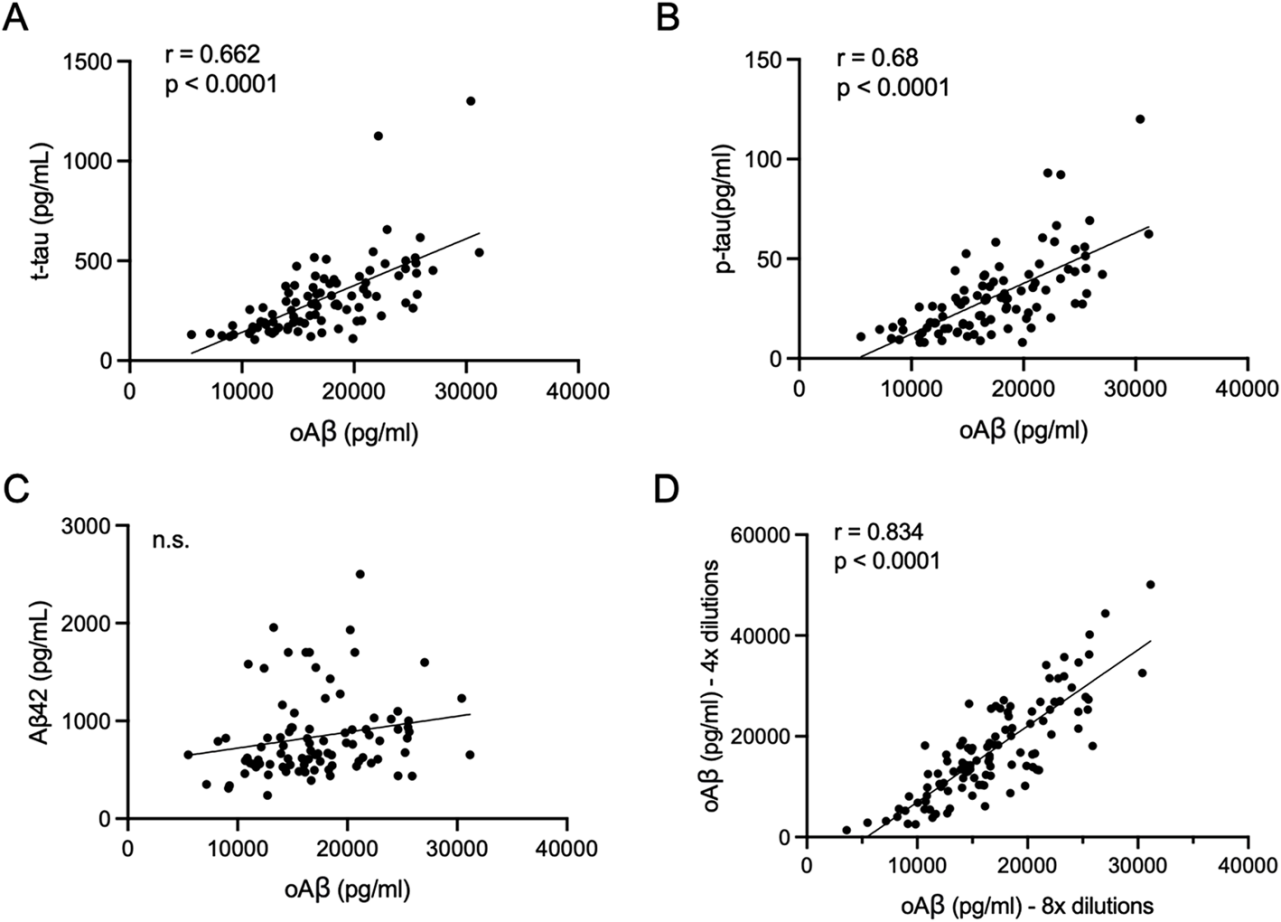
oAβ levels correlate significantly with tau and *p*-tau^181^ levels but not Aβ42 monomer levels. Human CSFs (*n* = 95) show highly significant positive correlations between 71A1/3D6 oAβ levels and total tau (t-Tau) levels (**A**) and phospho-tau (*p*-Tau^181^) levels (**B**) measured on the Alzheimer’s Disease Evaluation (ADEVL) platform at Mayo Clinic Laboratories. See also Supp Fig. 1. **C** Lack of correlation between 71A1/3D6 oAβ levels and Aβ1-42 monomer levels in the CSFs. **D** Reproducibility of plate-based 71A1/3D6 oAβ assay across two independent runs of another BWH cohort of patient CSFs (*n* = 108). Pearson correlation is used for all analyses

oAβ levels in the 108 CSF samples were quantified using the new 71A1/3D6 assay, yielding an average of 16,834.4 ± 5,356.1 pg/mL, with a range of 3,593.5 to 31,155.9 pg/mL (Fig. 6). In 95 of 108 subjects that also had CSF AD biomarkers done at Mayo Clinic Laboratories, we observed highly significant positive correlations between 71A1/3D6 signals and both total tau (311.3 ± 190.9 pg/mL) (*r* = 0.66, *p* < 0.0001) and phosho-T181) (30.7 ± 19.9 pg/mL) (*r* = 0.68, *p* < 0.0001; Pearson correlation coefficients) in the same CSFs (Fig. 6A, B). No statistically significant correlation between CSF oAβ levels and Aβ1-42 monomer levels (805.5 ± 385.4 pg/mL) was seen in these CSFs (Fig. 6C). In a subset of MAC CSFs (*n* = 11), the oAβ results of the new plate-based assay agreed well (*r* = 0.9091; *p* = 0.0003) with earlier results from its bead-based counterpart (Fig. 4C), in which we had previously reported similar positive correlations with t-Tau and p-Tau biomarkers measured by the ADmark (Athena Diagnostics) CSF assay in different BWH patients [17].

The 108 CSF samples were measured twice on two separate days (Fig. 6D) using either 4 × or 8 × dilutions. The two sets of measurements showed a strong correlation (*r* = 0.834, *p* < 0.0001), further supporting the assay’s stability and reproducibility.

Within the aforementioned MAC cohort (*n* = 108), plasma samples were also collected for 96 of the 108 patients at the time of LP. Measuring oAβ in those 96 matched plasmas showed no correlation between CSF and plasma oAβ values (data not shown). Moreover, no significant correlations were observed between plasma oAβ levels and AD CSF biomarkers (Aβ1-42, t-Tau, and p-Tau). Together, these results indicate that while oAβ levels in CSF show strong positive correlations with both t-tau and p-tau CSF biomarkers, plasma oAβ levels using this assay, while detectable, do not correlate with either CSF biomarker in the same patients.

## Discussion

The last two decades have witnessed a surge in studies of fluid biomarkers for Alzheimer’s disease. Major progress in identifying and validating CSF and plasma levels of Aβ monomers and numerous tau isoforms has led to their widespread inclusion in diagnostic algorithms and therapeutic screening for AD and related dementias. In striking contrast, diffusible oligomers/protofibrils of Aβ have achieved nowhere near a comparable level of quantitative analysis, despite their mechanistic importance in helping to initiate and drive both neuronal and microglial alterations in AD brain. Very few immunoassays that quantify oligomeric forms of Aβ in human biofluids have been systematically and rigorously validated.

In this study, we build on our published analysis [17] of a promising oligomer-preferring monoclonal antibody (71A1) having a unique Aβ 9–18 cyclized dimer as immunogen to improve upon its utility to quantify diffusible oAβ in aqueous brain extracts and human biofluids. We explore both the binding selectivity of 71A1 and its ability to neutralize oAβ-mediated synaptotoxicity. Using SPR in kinetic mode, we show that 71A1 exhibits a high affinity for soluble synthetic Aβ40(S26C)_2_ dimers that are covalently crosslinked to ensure they are dimeric during testing. This 71A1 binding was completely abolished by depolymerization to monomers with reducing agent. Likewise, 71A1 sensitively quantifies variably sized oligomers of synthetic Aβ42 (often referred to as ADDLs) that serve as a widely used standard for aqueously diffusible Aβ oligomers. Using SPR, we show that the oligomer selectivity of 71A1 resembles that of lecanemab, an approved therapeutic antibody that preferentially targets oligomeric (“protofibrillar”) Aβ with high affinity. In contrast, we found that donanemab, which specifically targets an N-terminal pyroglutamate-modified form of Aβ, displayed no binding by SPR to either Aβ40(S26C)_2_ or Aβ40 monomers, confirming the specificity of our 71A1 assay for wild-type Aβ.

Our SPR results revealed that 71A1 has a rapid association rate, although somewhat slower than that of lecanemab, and both antibodies exhibited slow dissociation rates, indicating a strong and relatively stable interaction with Aβ dimers. These findings suggest that 71A1 and lecanemab may share an ability to target the oligomeric forms of Aβ implicated in AD pathogenesis, given lecanemab’s successful clinical trials and its subsequent growing usage in treating MCI/mild AD patients in real-world settings.

Our new electrophysiological studies of 71A1 demonstrate the antibody’s ability to potently neutralize human brain oAβ-induced changes in neuronal excitatory/inhibitory balance and impairment of synaptic plasticity in hippocampal or cortical neurons (Fig. 1). The work indicates that the Aβ species recognized by 71A1 are neurotoxic, as evidenced by altered excitatory post-synaptic currents and calcium transients (Fig. 1). We examined the neuroactive potential of 71A1-purified oAβ species from aqueous AD brain extracts prepared without tissue homogenization to avoid breaking up amyloid plaque cores and vascular amyloid. The electrophysiological recordings in rat hippocampal neurons demonstrate that 71A1-purified oAβ significantly increases neuronal excitability. Specifically, we observed an increase in the amplitude of sEPSCs following both short (2-min) and long (30-min) exposure to diffusible AD oAβ, while the frequency of sEPSCs remained unchanged. Inhibitory postsynaptic currents were not significantly altered.

These results confirm the neuroactive properties of the oAβ species targeted by 71A1. Numerous studies have reported elevated neuronal activation in conditions associated with AD, including in cognitively normal carriers of the APOE4 allele [38–41], pre-symptomatic carriers of genetic mutations for familial AD [42], and patients with amnestic mild cognitive impairment (aMCI) [43–45]. In the case of early MCI, where memory deficits exceed what is expected for age, abnormally increased hippocampal activation has been proposed to serve compensatory roles by recruiting additional neural circuits [46, 47]. Excess activation may directly contribute to memory impairment and could be linked to widespread degenerative processes in prodromal AD [48].

Analysis of brain slices from a mutant APP knock-in model of AD revealed an age-dependent increase in 71A1-immunoreactive Aβ deposits, and this was paralleled by rising levels of aqueously soluble oAβ by the new 71A1/3D6 assay. Collectively, our findings provide evidence that 71A1 has potential applications not only in AD research and diagnostic monitoring but also in immunotherapeutic interventions for AD.

Of special interest is our analysis of the conditioned media of iPSC-derived human neurons that express APP or PS1 mutations. Previous studies demonstrate a robust relationship between these mutations and changes in the levels of secreted Aβ monomers in the respective iPSC-derived cultures [49–51]. However, previous studies investigating altered Aβ secretion have almost solely focused on monomeric forms due to the dearth of sensitive immunoassays that reliably quantify Aβ oligomers. With our development of an oligomer-preferring assay, we are now able to quantify pathological, aggregated Aβ species and determine how their levels change in the presence of various AD-causing mutations. Notably, the well-documented decrease of Aβ42 monomers in the case of the protective Icelandic APP mutation was associated with an analogous decrease in oAβ levels compared to isogenic control neurons. In contrast, both the Swedish APP and the PS1 M146V FAD-causing mutations substantially elevated oAβ levels in human neuronal media. These findings highlight the utility of the 71A1/3D6 assay in assessing wild-type vs. FAD-driven Aβ oligomer/protofibril generation.

The 71A1/3D6 plate-based assay first described and validated here improves upon our prior bead-based assay in several ways: greater stability by avoiding batch-to-batch bead variability; a more facile, operator-friendly workflow; resilience to repeated freeze–thaw cycles; smaller sample volumes; and less expense. Our assay improvements should help facilitate the further study of oAβ in human neuronal cultures, mouse brains, human brain tissue, and especially human biological fluids, offering a practical approach to monitor these hydrophobic neurotoxic species. In our analyses of cognitively impaired BWH clinic patients (the MAC Cohort) using the new plate-based assay, we obtained results consistent with our earlier findings using the bead-based format, namely, a highly significant positive correlation between oAβ levels and both p-Tau181 and total Tau levels in the CSF of cognitively symptomatic patients.

An important question for future work regards the proportion of pathologically relevant oAβ species, such as those we measured in CSF, that can reach plasma and be recovered by 71A1 in this far more complex protein- and lipid-rich matrix. Our blinded analyses of CSF-matched MAC cohort plasmas as well as a separate cohort of 199 plasma samples from untreated subjects in the A4 clinical trial population (not described in the current paper) revealed that plasma oAβ levels did not correlate with either CSF oAβ levels or CSF AD biomarkers nor with brain amyloid PET levels (data not shown). We previously conducted spike-and-recovery experiments by spiking human plasmas with soaking extracts or TBS homogenates of AD brain or with human CSF and then measuring the total recovery of oAβ in these spiked samples using the bead-based 71A1/3D6 assay. The results demonstrated mean recovery values within the acceptable range of 80–120% [17]. Although these data support the ability of the 71A1/3D6 assay to accurately quantify oAβ in plasma, it is likely that the rich macromolecular environment of plasma, containing a plethora of other hydrophobic components (e.g., lipids and lipoproteins), may complicate the measurement of plasma oAβ in a way that correlates with oAβ, total tau, and phosphorylated tau in CSF. Other studies have reported no correlation [52] or weak correlation [53, 54] between Aβ42/40 levels in plasma and CSF biomarkers, and also no correlation for t-tau between CSF and plasma [52–56]. However, a strong correlation has been observed for phosphorylated tau (pTau217, pTau181, pTau231) between matched plasma and CSF across entire cohorts [55, 56]. Notably, this correlation weakened [55] or was lost [56] when control samples are excluded and only AD patients are evaluated. We plan to enrich 71A1-immunoreactive oAβ species from human plasma to assess their molecular identity and the proteins and lipids that may bind and sequester them.

### Limitations

This study describes a technically improved assay for detecting diffusible oAβ and evaluates its performance using various biofluid samples. While the results are promising, some limitations should be noted. The sample size was sufficient for method development and initial validation but is limited for broader clinical application. Larger and more diverse cohorts will be needed to assess clinical utility, including effects of age, sex, and disease stage. We also observed modest variability in the absolute oAβ levels in about 25% of the individual biofluid samples when they are assayed repeatedly, which is relevant to the assay’s potential application to larger cohorts with greater sample heterogeneity. On the other hand, repeated assays of a single pool of 10–15 human plasmas showed low and acceptable CV% on both intra-plate (3–13% range over 10 days) and inter-date (mean of 13% across 10 days) (Table 2). The dynamic nature of oAβ, which could aggregate and disaggregate over time in some solutions, may affect assay values. The standard used for calibration (synthetic Aβ ADDLs) is an aggregated, heterogeneous protein mixture, which can also contribute to day-to-day differences in back-interpolated sample concentrations (Fig. 2C). As a result, the assay can show some instability in absolute values between runs across days. To address this, internal controls were included on each plate, and results were normalized accordingly. Still, it is important to be aware of this source of variability and always include internal controls and maintain assay protocol and reagents as consistently as possible to minimize bias.

## Conclusions

This study advances AD biomarker research by introducing a sensitive and stable plate-based assay for oAβ detection and quantification in human and mouse brain extracts, neuronal conditioned media, and human CSF. SPR demonstrates the preferential selectivity of 71A1 for Aβ oligomers over monomers. 71A1 binds strongly to Aβ40(S26C)_2_ dimers and ADDLs but has no significant binding to monomers. oAβ purified by 71A1 from AD brains abnormally increases hippocampal neuronal excitability, confirming that diffusible oAβ contributes to neuronal dysfunction. A plate-based 71A1/3D6 immunoassay sensitively quantifies oAβ in human brain extracts, CSF, and plasma. Aβ oligomer levels in CSF correlate positively with rising p-tau and total tau levels. 71A1-reactive oAβ levels are altered in iPSC-derived neurons carrying AD-relevant mutations, reflecting their abnormal Aβ42 production. Together, these findings validate 71A1 as a robust tool for isolating and quantifying oAβ, enabling new insights into the role of Aβ oligomers in AD. The 71A1/3D6 assay may advance diagnostic measurement and the monitoring of diffusible Aβ oligomers/protofibrils during AD-modifying treatments.

## Supplementary Information

Supplementary Material 1.

## Data Availability

No datasets were generated or analysed during the current study.

## Abbreviations

AD: Alzheimer's Disease
oAβ: Aβ oligomers
Aβ: Amyloid β-protein
CSF: Cerebrospinal fluid
SPR: Surface plasmon resonance
RB: Running buffer
aMCI: Amnestic mild cognitive impairment
ADDLs: Amyloid-β derived diffusible ligands
mAb: Monoclonal antibody
wt: Wild-type
PBS: Phosphate-buffered saline
TBS: Tris-buffered saline
sEPSCs: Spontaneous excitatory postsynaptic currents
sIPSCs: Spontaneous inhibitory postsynaptic currents
iPSC: Induced pluripotent stem cell
iNs: Induced neurons
IP: Immunoprecipitation
FAD: Familial Alzheimer’s Disease
CV: Coefficients of variation
βME: 2-Mercaptoethanol
MSD: Meso Scale Discovery
RT: Room temperature
LLoQ: The lower limit of reliable quantification
GuHCl: Guanidinium hydrochloride
SEC: Size exclusion chromatography
BWH: Brigham and Women’s Hospital
IRB: Institutional Review Board
MAC: BWH Memory and Aging Cohort
IHC: Immunohistochemistry
