# Supplementary material for: An improved immunoassay detects Aβ oligomers in human biofluids: their CSF levels rise with tau and phosphotau levels
Source: Alzheimers Res Ther. 2025 Jul 12;17:153. doi: 10.1186/s13195-025-01802-x (PMC12255133; doi:10.1186/s13195-025-01802-x)

**Supplemental Figure Legends**

**Supplemental Table 1 - Demographics of a BWH patient cohort (MAC)**

Data are presented as means ± standard error of the mean. CSF ADEVL data was not available for 13/108 patients. For 2 out of these 13 patients, gender and age were also not available.

**Supplemental Figure 1. Further analysis of plate-based 71A1/3D6 oAβ assay consistency to supplement Figures 4A and B.**

Correlation of 71A1/3D6 oAβ readouts from 8 plasmas (A) and 8 CSFs (B) across independent assay runs as graphed in Figures 4A and B but showing the individual comparisons of run 2 (x-axis) vs. run 3 (y-axis). The average CV% for the 8 CSF values across all 3 runs is 29%. The average CV% of the internal control CSF sample across all 3 runs is 21%. The average CV% for the 8 plasma values across all 3 runs is 18%, and the average CV% of the internal control plasma sample across all 3 runs is 9%.

**Supplemental Figure 2. oAβ and Aβ42 monomer levels in human neuron culture medium (CM) from iPSC-derived human neurons carrying endogenous or engineered mutations**

(A) Quantification of oAβ in CM from iPSC-derived models of FAD by the 71A1/3D6 plate-based assay. Values are measured to total protein levels in the lysates of each sample. (B) Aβ42 levels showed similar trends as oAβ levels. Neuronal CM were from an isogenic control line (grey); the Icelandic A673T/A673T APP protective mutation (red), the APP^swe^ mutation (light blue) and the APP^swe^PSEN1^M146V^/APP^swe^PSEN1^M146V^ mutations (dark blue). N = 2 differentiations, 3 independent wells per differentiation for each genotype. Each sample was measured in triplicate on the 71A1/3D6 assay. Data presented as mean values ± SD. ** p<0.01

**Supplemental Table 1**


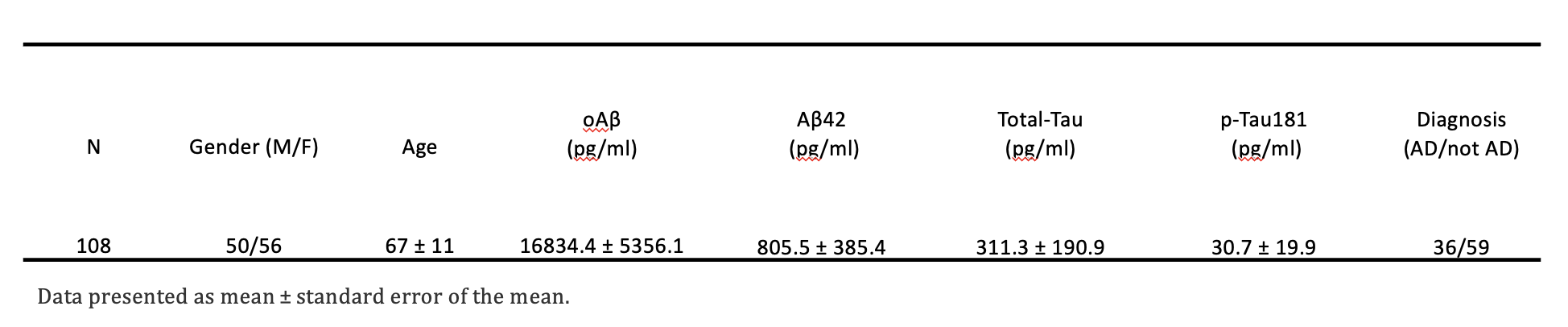


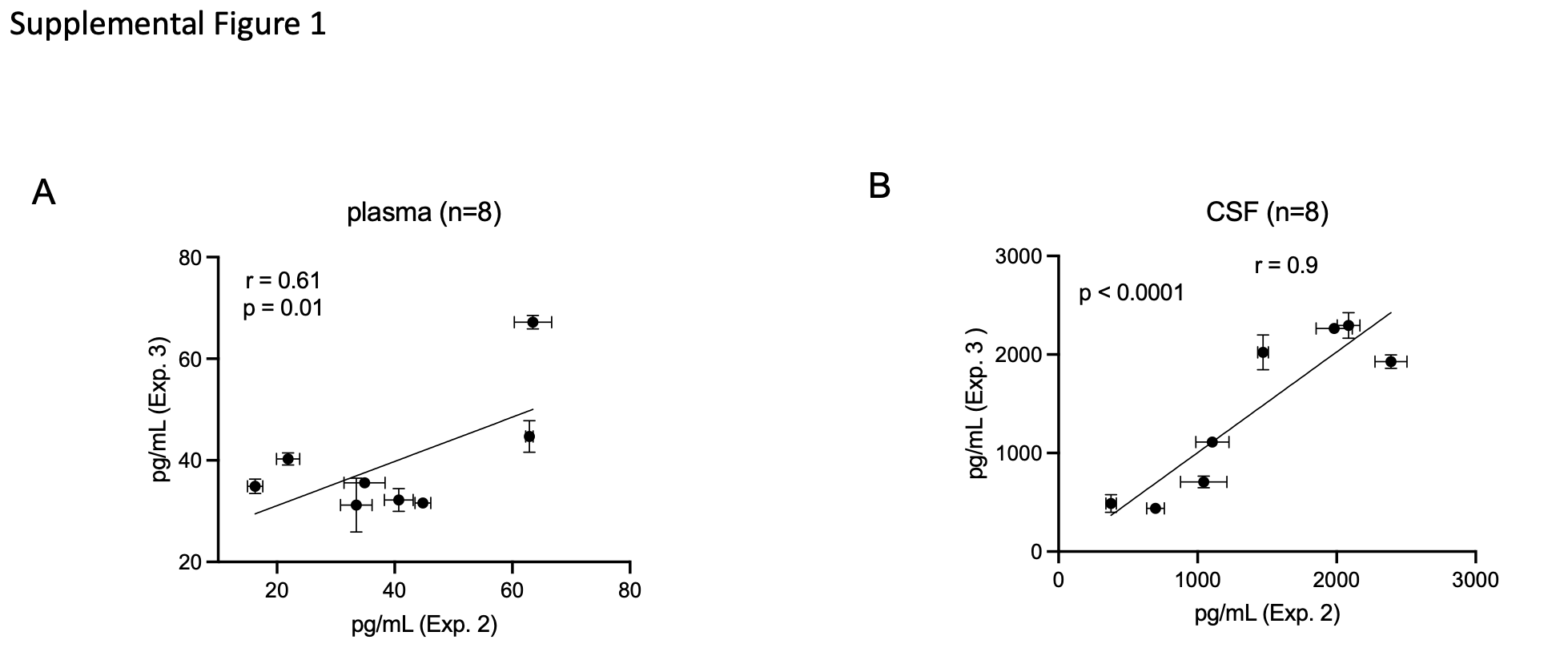


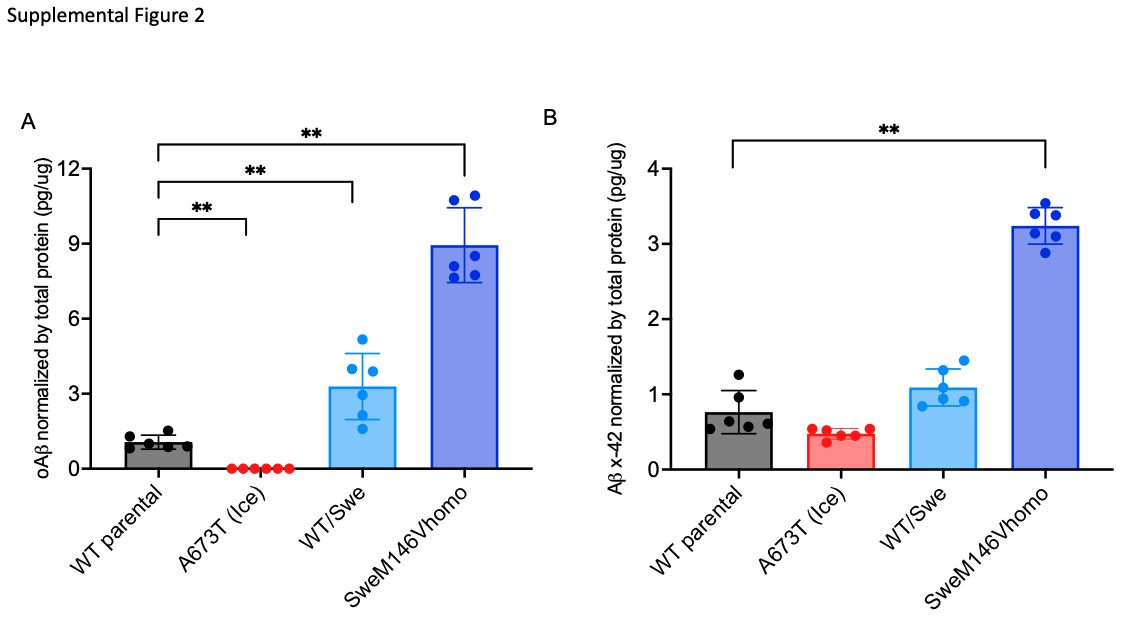

Supplement: Supplementary file 1 — Supplementary Material 1. [file 13195_2025_1802_MOESM1_ESM.docx]
